# Natural phytoalexin stilbene compound resveratrol and its derivatives as anti-tobacco mosaic virus and anti-phytopathogenic fungus agents

**DOI:** 10.1038/s41598-021-96069-1

**Published:** 2021-08-13

**Authors:** Pengfei Song, Xiuling Yu, Wenqiang Yang, Qingmin Wang

**Affiliations:** 1 College of Pharmacy, Linyi University, Linyi, 276000 People’s Republic of China; 2 State Key Laboratory of Elemento-Organic Chemistry, Research Institute of Elemento-Organic Chemistry, College of Chemistry, Nankai University, Tianjin, 300071 People’s Republic of China; 3 Collaborative Innovation Center of Chemical Science and Engineering (Tianjin), Tianjin, 300071 People’s Republic of China

**Keywords:** Chemical modification, Medicinal chemistry, Organic chemistry, Chemical synthesis

## Abstract

Plant diseases caused by plant viruses and pathogens seriously affect crop yield and quality, and it is very difficult to control them. The discovery of new leads based on natural products is an important way to innovate pesticides. Based on the resveratrol is a kind of natural phytoalexin, but it cannot be used as candidate for the development of new drug due to its poor druggability. The phenolic hydroxyl groups in the resveratrol structure are easily destroyed by oxidation, in order to improve its stability, ester formation is the most commonly used modification method in drug design. Their structures were characterized by ^1^H NMR, ^13^C NMR and HRMS. The activity against tobacco mosaic virus (TMV) of these ester derivatives has been tested for the first time. The bioassay results showed part of the target compounds exhibited good to excellent in vivo activities against TMV. The optimum compounds **III-2** (inhibitory rates of 50, 53, and 59% at 500 μg/mL for inactivation, curative, and protection activities in vivo, respectively), **III-4** (inhibitory rates of 57, 59, and 51% at 500 μg/mL, respectively), and **II-5** (inhibitory rates of 54, 52, and 51% at 500 μg/mL, respectively) displayed higher activity than commercial plant virucide ribavirin (inhibitory rates of 38, 37, and 40% at 500 μg/mL, respectively). Compounds **I-9** and **I-10** also showed excellent activities. The systematic study provides strong evidence that these simple resveratrol derivatives could become potential TMV inhibitors. The novel concise structure provides another new template for antiviral studies.

## Introduction

There are many different kinds of plant viruses, which have very extensive distribution; virus diseases rank only second to the fungi categories in agricultural plant diseases. Most commercial crops are subjected to production or quality decline because of the detriment of the plant virus. Plant virus are absolute parasitic in plant cells; the required material, energy, places are provided by the host cell. Because plants do not have the complete immune system, it is difficult to prevent plant virus disease completely, therefore plant virus disease is also known as the "plant cancer"^1^. Tobacco mosaic virus (TMV) was the first discovered plant virus and is widely used as a model virus in the study of new antiviral agents. Its host is very wide, and it can infect many plants including capsicum, cucumber, tomato, eggplant, solanum nigrum, and so on, which brings great harm to agricultural production^2^. Although several commercial antiviral agents against TMV have been used, efficient and practical varieties are few. The most widely used antiviral agent ribavirin only gave an inhibition rate of no more than 50% anti-TMV effect at 500 μg/mL, and the most effective antiviral agent ningnanmycin has an inhibitory effect of 50–60% at 500 μg/mL. Plant diseases caused by TMV are difficult to control. Therefore, it is urgent to develop novel antiviral agents with a simple structure and outstanding activities^3^.

**Figure 1:**
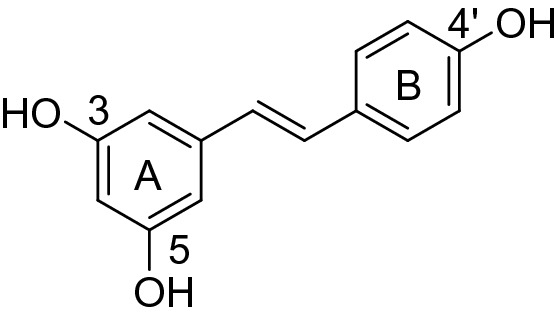
Structure of resveratrol.

Lead discovery and optimization based on natural products are crucial means to develop novel pesticides, because their novel scaffolds can provide modes of action different from those of existing agents. Some natural products have been commercialized or used as lead compounds for plant-virus control; however, there are only a few reported economically viable antiviral chemicals available for practical application in plant protection. The naturally occurring resveratrol (3,5,4′-trihydroxy-trans-stilbene, Fig. 1) is a phytoalexin which can be activated by adverse conditions of plants, protecting against fungal infections^4–11^. First isolated in 1940 from *Veratrum grandiflorum* by Takaoka^12,13^, and later, it has been obtained in larger quantities from the roots of *Polygonum cuspidatum*^14^, a plant used in traditional Chinese medicine. It attracted wider attention only in 1992 when its presence in wine was suggested as the explanation for cardioprotective effects^15^. Resveratrol is the most representative compound of stilbene analogues, resveratrol and its derivatives exhibits a wide range of intriguing biological activities, such as antibacterial^16^, antitumour^17,18^, antiviral^19^, antioxidant^20^, and antihypertensive activities^21^. Furthermore, stilbene moieties may have numerous agrochemical applications, such as in herbicides^22–25^. Recently, considerable attention has been focused on resveratrol derivatives. Although resveratrol possesses a series of pharmacological activities, its applications in the field of pesticide development have not been reported.

Studies have shown that modification of the structure of natural compounds can improve their biological activities. To improve the stability of resveratrol, many researchers have undertaken the synthesis and activity evaluation of resveratrol derivatives and analogues. They have modified the phenolic hydroxyl groups, double bonds and benzene ring of resveratrol so as to further understand the interactions among functional groups and its structure–activity relationship. Grow evidence indicates that this compound could be used as a lead compound in the design of drugs and resveratrol derivatives synthesized from the lead compound resveratrol or stilbene have a higher efficacy and lower toxicity^26–29^.

**Figure 2:**
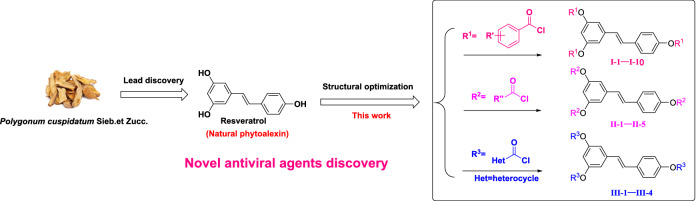
The design and synthesis of compounds **I-1–I-10, II-1–II-5** and **III-1–III-4**.

Whilst the antiviral activity of resveratrol has been extensively studied, little is known about the activity against plant viruses of resveratrol and resveratrol derivatives. Ester formation is the most commonly used modification method in drug design. A few resveratrol ester derivatives had been synthesized and tested for their antitumor activity. However, reports of the anti-TMV activity of the resveratrol ester derivatives are rather rare, and no examples are documented in the recent literature. In this paper, in order to increase the stability and druggability of resveratrol, we synthesized a series of resveratrol ester derivatives and their activities against TMV were evaluated (Fig. 2). The structure–activity relationships of these derivatives were discussed as well. Additionally, the synthesized derivatives were also investigated for their potential as fungicidal, or insecticidal agents. Resveratrol derivatives will become a research focus in future in agricultural applications.

## Materials and methods

### Instruments

All other commercial reagents and solvents were used as received without further purification. Reaction solvents were distilled from calcium hydride for dichloromethane and from sodium metal and benzophenone for tetrahydrofuran. E-resveratrol (98%) was purchased from Shanxi Sciphar Hi-tech Industry Co., Ltd. (Shanxi, China). Reaction progress was monitored by thin-layer chromatography on silica gel GF254 with detection by ultraviolet (UV). Melting points were obtained using an X-4 binocular microscope melting point (mp) apparatus and are uncorrected. Yields were not optimized. ^1^H-NMR spectra and ^13^C-NMR spectra were recorded utilizing a Bruker AV400 spectrometer with CDCl_3_ as solvent and tetramethylsilane as internal standard. Chemical shifts (δ) were given in parts per million (ppm). High-resolution mass spectra (HRMS) data were obtained with a Fourier transform ion cyclotron resonance mass spectrometry (FTICR-MS) spectrometer (ionspec, 7.0 T).

### General synthetic procedure for the target compounds I-1–I-10, II-1–II-5, and III-1–III-4 (Scheme 1)

**Scheme 1:**
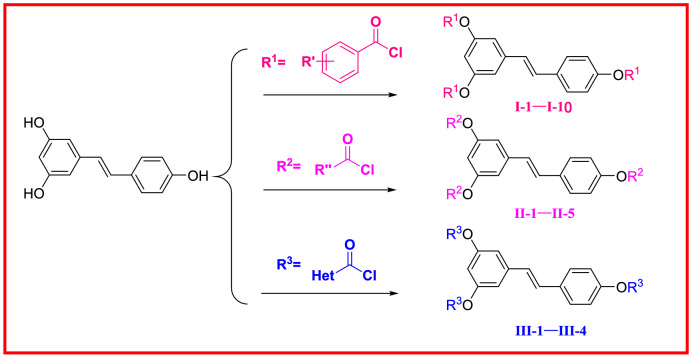
The synthetic route of target compounds.

E-resveratrol (0.1 g, 0.44 mmol) was dissolved in dichloromethane (20 mL), and Et_3_N (0.22 g, 2.19 mmol) was added. Then, PhCOCl (0.31 g, 2.19 mmol) were slowly added and the reaction mixture was stirred at room temperature. The progress of the reaction was monitored by TLC until the reaction was complete. Then the reaction mixture was added dichloromethane and water. The aqueous phase was separated and then extracted with dichloromethane twice. The combined organic layer was washed with saturated brine, dried over anhydrous sodium sulfate, and filtered. After the solvent was removed in vacuo, the residue was purified by column chromatography on silica gel to give the target compound **I-1** as white solid.

**Scheme 2:**
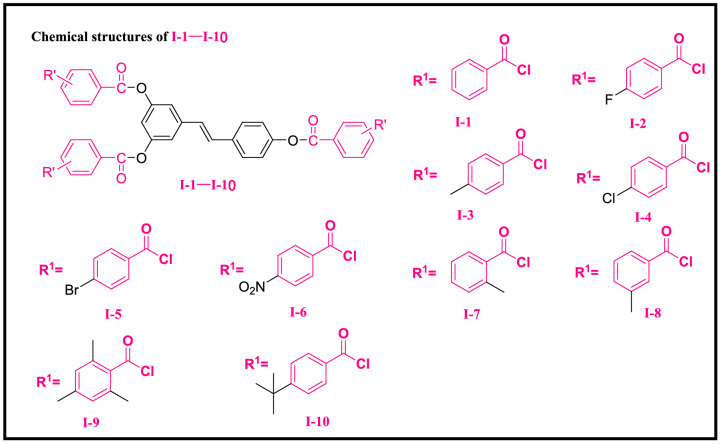
Chemical structures of **I-1–I-10**.

**Scheme 3:**
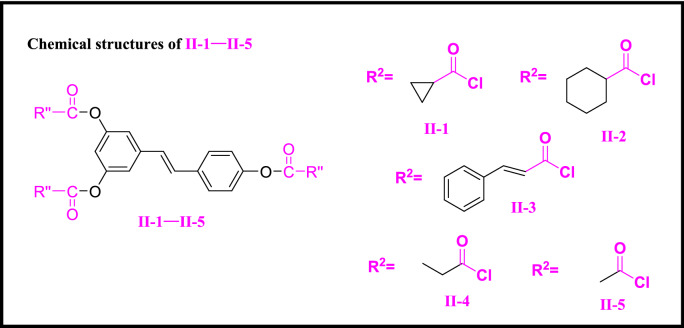
Chemical structures of **II-1–II-5**.

**Scheme 4:**
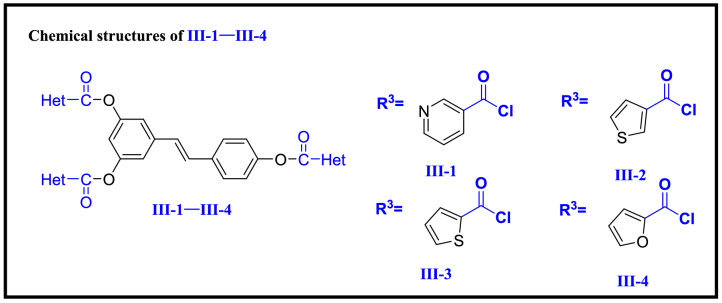
Chemical structures of **III-1–III-4**.

Compounds **I-2–I-10** (Scheme 2), **II-1–II-5** (Scheme 3) and **III-1–III-4** (Scheme 4) were synthesized according to the method used for compound **I-1**.

*Data for* (E)-5-(4-(benzoyloxy)styryl)-1,3-phenylene dibenzoate (**I-1**). White solid; yield 96%; mp 170–172 °C. ^1^H NMR (400 MHz, CDCl_3_) δ8.22 (m, 6H), 7.71–7.61 (m, 3H), 7.60–7.48 (m, 8H), 7.36–7.30 (m, 2H), 7.15 (m, 5H); ^13^C NMR (100 MHz, CDCl_3_) δ165.13, 164.86, 151.70, 150.73, 139.75, 134.56, 133.83, 133.70, 130.27, 130.23, 129.80, 129.45, 129.25, 128.68, 128.63, 127.78, 127.27, 122.09, 117.24, 114.82; HRMS (ESI) (m/z): calcd for C_35_H_24_O_6_ [M + NH_4_]^+^558.1911, found 558.1911.

*Data for* (E)-5-(4-((4-fluorobenzoyl)oxy)styryl)-1,3-phenylene bis(4-fluorobenzoate) (**I-2**). White solid; yield 81%; mp 167–169 °C. ^1^H NMR (400 MHz, CDCl_3_) δ8.28–8.20 (m, 6H), 7.56 (d, *J* = 8.0 Hz, 2H), 7.31 (d, *J* = 2.0 Hz, 2H), 7.25–7.17 (m, 8H), 7.15–7.04 (m, 3H); ^13^C NMR (100 MHz, CDCl_3_) δ164.45 (d, *J* = 119 Hz, 1C), 164.14, 151.56, 150.62, 139.80, 134.59, 132.89 (d, *J* = 9 Hz, 1C), 132.90, 132.80, 129.87, 127.80, 127.24, 125.47, 122.05, 117.24, 116.05, 115.97, 115.83, 115.74, 114.72; HRMS (ESI) (m/z): calcd for C_35_H_21_F_3_O_6_ [M + NH_4_]^+^ 612.1628, found 612.1630.

*Data for* (E)-5-(4-((4-methylbenzoyl)oxy)styryl)-1,3-phenylene bis(4-methylbenzoate) (**I-3**). White solid; yield 80%; mp 159–161 °C. ^1^H NMR (400 MHz, CDCl_3_) δ8.14–8.07 (m, 6H), 7.55 (d, *J* = 8.4 Hz, 2H), 7.36–7.28 (m, 8H), 7.24–7.02 (m, 5H), 2.46 (s, 9H); ^13^C NMR (100 MHz, CDCl_3_) δ165.16, 164.89, 151.76, 150.79, 144.64, 144.50, 139.66, 134.50, 130.29, 130.26, 129.73, 129.38, 129.32, 127.72, 127.30, 126.72, 126.54, 122.10, 117.16, 114.86, 21.80; HRMS (ESI) (m/z): calcd for C_38_H_30_O_6_ [M + NH_4_]^+^ 600.2381, found 600.2379.

*Data for* (E)-5-(4-((4-chlorobenzoyl)oxy)styryl)-1,3-phenylene bis(4-chlorobenzoate) (**I-4**). White solid; yield 97%; mp 165–167 °C. ^1^H NMR (400 MHz, CDCl_3_) δ8.14–8.11 (m, 4H), 8.07 (d, *J* = 8.4 Hz, 2H), 7.55–7.47 (m, 9H), 7.30 (d, *J* = 2.0 Hz, 2H), 7.21 (d, *J* = 8.4 Hz, 2H), 7.13–7.04 (m, 2H); ^13^C NMR (100 MHz, CDCl_3_) δ164.24, 163.96, 161.30, 151.51, 150.58, 141.42, 140.43, 140.26, 139.83, 134.61, 131.89, 131.60, 129.89, 129.38, 129.08, 129.00, 127.81, 122.00, 117.23, 114.62; HRMS (ESI) (m/z): calcd for C_35_H_21_Cl_3_O_6_ [M + NH_4_]^+^ 660.0742, found 660.0731.

*Data for* (E)-5-(4-((4-bromobenzoyl)oxy)styryl)-1,3-phenylene bis(4-bromobenzoate) (**I-5**). White solid; yield 85%; mp 183–185 °C. ^1^H NMR (400 MHz, CDCl_3_) δ8.08–8.05 (m, 4H), 7.99 (d, *J* = 8.0 Hz, 2H), 7.68–7.65 (m, 6H), 7.55 (d, *J* = 8.4 Hz, 2H), 7.31 (d, *J* = 2.0 Hz, 2H), 7.22 (d, *J* = 8.4 Hz, 2H), 7.17–7.03 (m, 3H); ^13^C NMR (100 MHz, CDCl_3_) δ164.43, 164.13, 161.46, 151.48, 150.55, 139.84, 134.61, 132.41, 132.10, 132.01, 131.95, 131.70, 129.90, 129.18, 128.08, 127.82, 127.20, 122.01, 117.25, 114.63; HRMS (ESI) (m/z): calcd for C_35_H_21_Br_3_O_6_[M + NH_4_]^+^793.9206, found 793.9171.

*Data for* (E)-5-(4-((4-nitrobenzoyl)oxy)styryl)-1,3-phenylene bis(4-nitrobenzoate) (**I-6**). Pale yellow solid; yield 79%; mp 237–239 °C. ^1^H NMR (400 MHz, CDCl_3_) δ8.42–8.27 (m, 12H), 7.59 (d, *J* = 8.4 Hz, 2H), 7.38 (d, *J* = 2.0 Hz, 2H), 7.27 (d, *J* = 8.0 Hz, 2H), 7.23–7.06 (m, 3H); ^13^C NMR (100 MHz, CDCl_3_) δ162.97, 151.28, 151.09, 150.39, 140.11, 134.79, 134.46, 131.40, 131.37, 130.95, 130.20, 128.86, 127.97, 127.15, 123.89, 123.80, 123.72, 121.91, 117.40, 114.37; HRMS (ESI) (m/z): calcd for C_35_H_21_N_3_O_12_ [M-OH]^-^ 658.1092, found 657.9651.

*Data for* (E)-5-(4-((2-methylbenzoyl)oxy)styryl)-1,3-phenylene bis(2-methylbenzoate) (**I-7**). White solid; yield 81%; mp 192–193 °C. ^1^H NMR (400 MHz, CDCl_3_) δ8.21–8.15 (m, 3H), 7.57 (d, *J* = 8.4 Hz, 2H), 7.52–7.47 (m, 3H), 7.36–7.31 (m, 8H), 7.22 (d, *J* = 8.4 Hz, 2H), 7.15–7.05 (m, 3H), 2.70 (s, 6H), 2.68 (s, 3H); ^13^C NMR (100 MHz, CDCl_3_) δ165.74, 165.38, 151.70, 150.68, 141.57, 141.43, 139.72, 134.54, 132.96, 132.83, 132.06, 132.01, 131.30, 131.21, 129.75, 128.18, 127.75, 127.29, 126.02, 125.96, 122.19, 117.26, 114.98, 22.07, 22.00; HRMS (ESI) (m/z): calcd for C_38_H_30_O_6_ [M + NH_4_]^+^ 600.2381, found 600.2379.

*Data for* (E)-5-(4-((3-methylbenzoyl)oxy)styryl)-1,3-phenylene bis(3-methylbenzoate) (**I-8**). White solid; yield 78%; mp 107–108 °C. ^1^H NMR (400 MHz, CDCl_3_) δ8.04–8.00 (m, 6H), 7.56 (d, *J* = 8.4 Hz, 2H), 7.47–7.38 (m, 6H), 7.32 (d, *J* = 2.0 Hz, 2H), 7.23 (t, *J* = 8.0 Hz, 2H), 7.18–7.04 (m, 3H), 2.46 (s, 6H), 2.45 (s, 3H); ^13^C NMR (100 MHz, CDCl_3_) δ165.27, 164.98, 151.77, 150.80, 139.72, 138.51, 138.46, 134.56, 134.44, 130.77, 130.72, 129.78, 129.41, 129.23, 128.55, 128.50, 127.75, 127.42, 127.37, 127.32, 122.08, 117.17, 114.80, 21.32; HRMS (ESI) (m/z): calcd for C_38_H_30_O_6_ [M + NH_4_]^+^ 600.2381, found 600.2379.

*Data for* (E)-5-(4-((2,4,6-trimethylbenzoyl)oxy)styryl)-1,3-phenylene bis(2,4,6-trimethylbenzoate) (**I-9**). White solid; yield 79%; mp 173–175 °C. ^1^H NMR (400 MHz, CDCl_3_) δ7.59 (d, *J* = 8.4 Hz, 2H), 7.33 (d, *J* = 1.6 Hz, 2H), 7.27–7.24 (m, 2H), 7.17–7.06 (m, 3H), 6.95–6.91 (m, 6H), 2.48 (s, 12H), 2.46 (s, 6H), 2.33 (s, 6H), 2.32 (s, 3H); ^13^C NMR (100 MHz, CDCl_3_) δ168.31, 167.88, 151.62, 150.56, 140.27, 140.09, 139.80, 135.93, 135.69, 134.62, 129.85, 129.51, 128.79, 128.70, 127.86, 127.33, 121.95, 117.04, 114.26, 21.23, 20.24, 20.07; HRMS (ESI) (m/z): calcd for C_44_H_42_O_6_ [M + NH_4_]^+^ 684.3320, found 684.3316.

*Data for* (E)-5-(4-((4-(tert-butyl)benzoyl)oxy)styryl)-1,3-phenylene bis(4-(tert-butyl)benzoate) (**I-10**). Pale yellow solid; yield 74%; mp 102–103 °C. ^1^H NMR (400 MHz, CDCl_3_) δ8.16–8.12 (m, 6H), 7.57–7.52 (m, 8H), 7.30 (d, *J* = 2.0 Hz, 2H), 7.21 (d, *J* = 8.6 Hz, 2H), 7.14–7.02 (m, 3H), 1.38 (s, 18H), 1.37 (s, 9H); ^13^C NMR (100 MHz, CDCl_3_) δ165.11, 164.86, 157.62, 157.49, 151.78, 150.80, 139.66, 134.51, 130.18, 130.14, 129.74, 127.74, 127.30, 126.66, 126.47, 125.67, 125.61, 122.12, 117.19, 114.87, 35.25, 31.14; HRMS (ESI) (m/z): calcd for C_47_H_48_O_6_ [M + NH_4_]^+^ 726.3789, found 726.4049.

*Data for* (E)-5-(4-((cyclopropanecarbonyl)oxy)styryl)-1,3-phenylene dicyclopropanecarboxylate (**II-1**). Yellow solid; yield 71%; mp 136–138 °C. ^1^H NMR (400 MHz, CDCl_3_) δ7.47 (d, *J* = 8.4 Hz, 2H), 7.13–6.91 (m, 6H), 6.83 (t, *J* = 2.0 Hz, 1H), 1.89–1.79 (m, 3H), 1.22–1.14 (m, 6H), 1.07–0.99 (m, 6H); ^13^C NMR (100 MHz, CDCl_3_) δ173.44, 173.11, 151.40, 150.52, 139.42, 134.36, 129.55, 127.59, 127.19, 121.92, 116.82, 114.50, 13.04, 9.40, 9.35; HRMS (ESI) (m/z): calcd for C_26_H_24_O_6_ [M + NH_4_]^+^ 450.1911, found 450.1914.

*Data for* (E)-5-(4-((cyclohexanecarbonyl)oxy)styryl)-1,3-phenylene dicyclohexanecarboxylate (**II-2**). White solid; yield 72%; mp 131–133 °C. ^1^H NMR (400 MHz, CDCl_3_) δ7.47 (d, *J* = 8.4 Hz, 2H), 7.08 (.m, *J* = 1.6 Hz, 3H), 7.04 (d, *J* = 5.12 Hz, 2H), 7.00–6.91 (m, 1H), 6.82–6.72 (m, 1H), 2.60–2.50 (m, 3H), 2.12–2.02 (m, 6H), 1.87–1.78 (m, 6H), 1.71–1.68 (m, 2H), 1.65–1.55 (m, 6H), 1.43–1.24 (m, 10H); ^13^C NMR (100 MHz, CDCl_3_) δ174.47, 174.11, 151.56, 150.65, 139.44, 134.34, 129.55, 127.58, 127.20, 121.88, 116.73, 114.42, 43.22, 28.94, 25.72, 25.36; HRMS (ESI) (m/z): calcd for C_35_H_42_O_6_ [M + NH_4_]^+^ 576.3320, found 576.3323.

*Data for* 5-((E)-4-(cinnamoyloxy)styryl)-1,3-phenylene (2E,2′E)-bis(3-phenylacrylate) (**II-3**). Yellow solid; yield 83%; mp 87–89 °C. ^1^H NMR (400 MHz, CDCl_3_) δ7.93–7.86 (m, 3H), 7.62–7.57 (m, 6H), 7.53 (d, *J* = 8.6 Hz, 2H), 7.46–7.38 (m, 10H), 7.25 (d, *J* = 2.3 Hz, 2H), 7.18 (d, *J* = 8.6 Hz, 2H), 7.15–6.98 (m, 3H), 6.67–6.61 (m, 3H); ^13^C NMR (100 MHz, CDCl_3_) δ165.36, 165.04, 151.52, 150.57, 147.07, 146.80, 139.62, 134.51, 134.16, 134.11, 130.86, 130.79, 129.70, 129.06, 128.98, 128.41, 128.36, 127.73, 127.30, 121.98, 117.19, 117.01, 114.58; HRMS (ESI) (m/z): calcd for C_41_H_30_O_6_ [M + NH_4_]^+^ 636.2381, found 636.2383.

*Data for* (E)-5-(4-(propionyloxy)styryl)-1,3-phenylene dipropionate (**II-4**). Brown solid; yield 89%; mp 56–58 °C. ^1^H NMR (400 MHz, CDCl_3_) δ7.48 (d, *J* = 8.4 Hz, 2H), 7.11 (d, *J* = 2.0 Hz, 2H), 7.10–7.07 (m, 2H), 7.05–6.92 (m, 2H), 6.82 (t, *J* = 2.0 Hz, 1H), 2.63–2.56 (m, 6H), 1.27 (t, *J* = 7.6 Hz, 9H); ^13^C NMR (100 MHz, CDCl_3_) δ172.92, 172.57, 151.41, 150.52, 139.50, 134.39, 129.60, 127.63, 127.20, 121.89, 116.82, 114.41, 27.78, 9.08, 9.05; HRMS (ESI) (m/z): calcd for C_23_H_24_O_6_ [M + NH_4_]^+^ 414.1911, found 414.1910.

*Data for* (E)-5-(4-acetoxystyryl)-1,3-phenylene diacetate (**II-5**). White solid; yield 65%; mp 186–188 °C. ^1^H NMR (400 MHz, CDCl_3_) δ7.48 (d, *J* = 8.5 Hz, 2H), 7.13–7.11 (m, 2H), 7.10–7.07 (m, 2H), 7.05–6.93 (m, 2H), 6.84–6.81 (m, 1H), 2.31 (s, 9H); ^13^C NMR (100 MHz, CDCl_3_) δ169.45, 169.04, 151.32, 150.44, 139.55, 134.48, 129.67, 127.68, 127.20, 121.91, 116.93, 114.42, 21.14; HRMS (ESI) (m/z): calcd for C_20_H_18_O_6_ [M + NH_4_]^+^ 372.1442, found 372.1443.

*Data for* (E)-5-(4-(nicotinoyloxy)styryl)-1,3-phenylene dinicotinate (**III-1**). Yellow solid; yield 71%; mp 135–137 °C. ^1^H NMR (400 MHz, CDCl_3_) δ9.45–9.39 (m, 3H), 8.92–8.85 (m, 3H), 8.51–8.44 (m, 3H), 7.58 (d, *J* = 8.4 Hz, 1H), 7.54–7.47 (m, 4H), 7.37 (d, *J* = 1.6 Hz, 1H), 7.25–7.20 (m, 2H), 7.17–7.14 (m, 1H), 7.12–6.92 (m, 3H); ^13^C NMR (100 MHz, CDCl_3_) δ163.79, 163.49, 154.18, 151.36, 151.26, 137.77, 134.74, 130.08, 127.91, 127.76, 127.19, 125.33, 123.65, 123.60, 121.96, 121.85, 117.37; HRMS (ESI) (m/z): calcd for C_32_H_21_N_3_O_6_ [M + H]^+^ 544.1503, found 544.1505.

*Data for* (E)-5-(4-((thiophene-2-carbonyl)oxy)styryl)-1,3-phenylene bis(thiophene-3-carboxylate) (**III-2**). Brown solid; yield 70%; mp 138–140 °C. ^1^H NMR (400 MHz, CDCl_3_) δ8.34–8.29 (m, 3H), 7.69–7.65 (m, 3H), 7.53 (d, *J* = 8.6 Hz, 2H), 7.40–7.36 (m, 3H), 7.29 (d, *J* = 2.0 Hz, 2H), 7.20 (d, *J* = 8.6 Hz, 2H), 7.16–7.01 (m, 3H); ^13^C NMR (100 MHz, CDCl_3_) δ160.96, 160.65, 151.42, 150.47, 139.70, 134.54, 134.35, 134.17, 132.78, 132.56, 129.77, 128.24, 127.76, 127.26, 126.55, 126.45, 122.06, 117.19, 114.74; HRMS (ESI) (m/z): calcd for C_29_H_18_O_6_S_3_ [M + NH_4_]^+^ 576.0604, found 576.0605.

*Data for* (E)-5-(4-((thiophene-2-carbonyl)oxy)styryl)-1,3-phenylene bis(thiophene-2-carboxylate) (**III-3**). Yellow solid; yield 78%; mp 113–115 °C. ^1^H NMR (400 MHz, CDCl_3_) δ8.03–7.96 (m, 3H), 7.71–7.65 (m, 3H), 7.54 (d, *J* = 8.3 Hz, 2H), 7.34–7.29 (m, 2H), 7.23 (d, *J* = 8.4 Hz, 2H), 7.20–7.16 (m, 3H), 7.13–7.01 (m, 3H); ^13^C NMR (100 MHz, CDCl_3_) δ160.50, 160.19, 151.27, 150.37, 139.70, 134.99, 134.81, 134.61, 133.88, 133.64, 132.78, 132.52, 129.84, 128.15, 128.09, 127.76, 127.25, 122.01, 117.22, 114.62; HRMS (ESI) (m/z): calcd for C_29_H_18_O_6_S_3_ [M + NH_4_]^+^ 576.0604, found 576.0605.

*Data for* (E)-5-(4-((furan-2-carbonyl)oxy)styryl)-1,3-phenylene bis(furan-2-carboxylate) (**III-4**). Brown solid; yield 85%; mp 144–146 °C. ^1^H NMR (400 MHz, CDCl_3_) δ7.73–7.66 (m, 3H), 7.53 (d, *J* = 7.9 Hz, 2H), 7.43–7.37 (m, 3H), 7.32–7.28 (m, 2H), 7.22 (d, *J* = 8.1 Hz, 2H), 7.15–6.98 (m, 3H), 6.64–6.56 (m, 3H); ^13^C NMR (100 MHz, CDCl_3_) δ156.83, 156.46, 150.92, 149.99, 147.46, 147.27, 143.89, 143.68, 139.76, 134.65, 129.88, 127.81, 127.23, 121.95, 119.87, 119.63, 117.21, 114.48, 112.32, 112.26; HRMS (ESI) (m/z): calcd for C_29_H_18_O_9_ [M + NH_4_]^+^ 528.1289, found 528.1291.

### Biological assay

Each bioassay was repeated three times at 25 ± 1 °C. The activity results were estimated according to a percentage scale of 0–100 (0 indicating no activity and 100 indicating total mortality). The bioassay procedures for the anti-TMV, fungicidal, and insecticidal activities of the synthesized compounds are described in detailed in our published literature and can also be found in the “Supporting Information S1”^30^.

## Results and discussion

### Synthesis

In order to investigate structure–activity relationships (SARs), resveratrol was chosen as a precursor according to the in vivo anti-TMV activity listed in Table 1. Several series of resveratrol derivatives **I-1–I-10**, **II-1–II-5** and **III-1–III-4** were synthesized according to procedures in Scheme 1. As shown in Scheme 1, commercially available E-resveratrol was reacted with corresponding aryl chloride, alkyl chloride and heterocyclic chloride to give resveratrol esters **I-1–I-10**, **II-1–II-5** and **III-1–III-4** in good yields. The detailed procedure is given in “Materials and methods”. It’s a simple route for the preparation of resveratrol derivatives with favorable yield.

**Table 1:** The anti-TMV activity of **I-1–I-10**, **II-1–II-5** and **III-1–III-4.**

Compounds	Concentration (μg/mL)	Relative inhibition rate (%)^a^		
		Inactivation effect	Curative effect	Protection effect
**I-1**	500	32 ± 2		
**I-2**	500	36 ± 1		
**I-3**	500	34 ± 3		
**I-4**	500	35 ± 3		
**I-5**	500	42 ± 4		
**I-6**	500	38 ± 2		
**I-7**	500	37 ± 2		
**I-8**	500	38 ± 3		
**I-9**	*500*	*54 ± 1*	*54 ± 4*	*55 ± 3*
	*100*	*24 ± 1*	*15 ± 1*	*20 ± 1*
**I-10**	*500*	*51 ± 1*	*56 ± 2*	*47 ± 2*
	*100*	*21 ± 1*	*18 ± 2*	*17 ± 2*
**II-1**	500	23 ± 5		
**II-2**	500	43 ± 1	44 ± 4	40 ± 2
	100	24 ± 2	15 ± 1	0
**II-3**	500	39 ± 3		
**II-4**	500	41 ± 5	43 ± 2	46 ± 2
	100	26 ± 1	0	25 ± 1
**II-5**	*500*	*54 ± 1*	*52 ± 2*	*51 ± 4*
	*100*	*25 ± 2*	*28 ± 1*	*23 ± 1*
**III-1**	500	30 ± 2		
**III-2**	*500*	*50 ± 1*	*53 ± 1*	*59 ± 4*
	*100*	*25 ± 2*	*20 ± 2*	*28 ± 1*
**III-3**	500	38 ± 3		
**III-4**	*500*	*57 ± 1*	*59 ± 3*	*51 ± 4*
	*100*	*20 ± 2*	*26 ± 1*	*21 ± 1*
Resveratrol^b^	500	50 ± 1	43 ± 2	45 ± 2
	100	13 ± 1	15 ± 1	18 ± 1
Ribavirin^b^	500	38 ± 2	37 ± 1	40 ± 1
	100	11 ± 2	13 ± 1	12 ± 1

^a^Average of three replicates. All results are expressed as the mean ± standard deviation (SD). Prominent activity data were presented in italics.

^b^Positive control.

### Phytotoxic activity

Phytotoxic activity (according to the criterion of safety evaluation of pesticide to crops, NYT 1965.1-2010) of compounds **I-1–I-10**, **II-1–II-5** and **III-1–III-4** against tobacco was first tested. The data of phytotoxic activity at 500 μg/mL indicated that all of the compounds **I-1–I-10**, **II-1–II-5** and **III-1–III-4** showed no toxicity to the tested plant.

### Antiviral activity

Compounds **I-1–I-10**, **II-1–II-5** and **III-1–III-4** were evaluated for their antiviral activity against TMV. The results of anti-TMV activities of these derivatives are listed in Table 1. To make a judgment on the antiviral potency of the synthesized compounds, the commercially available plant virucide ribavirin and lead compound resveratrol were used as the controls. All of the compounds were tested at both 500 and 100 μg/mL.

The synthesized compounds showed similar or higher in vivo activity against TMV than the commercial plant virucide ribavirin. At the concentration of 500 μg/mL, resveratrol ester derivative containing meta-thienyl moieties **III-2** displayed the best inhibitory effect of inactivation activity, curative activity, and protection activity with values of 50, 53, and 59%, respectively. Ribavirin as a control was studied at the same conditions with values of 38, 37, and 40%, respectively. In contrast to the experimental data, the results indicated that compound **III-2** was more efficient than ribavirin in vivo activity against TMV. At the concentration of 100 μg/mL, **III-2** displayed inactivation activity, 25%; curative activity, 20%; and protection activity, 28%; which is higher than that of ribavirin (inactivation activity, 11%; curative activity, 13%; and protection activity, 12%). Resveratrol ester derivative **III-4** bearing furan groups also gave relatively higher activity (57, 59, and 51% at 500 μg/mL) than ribavirin. Especially the curative activity at 500 μg/mL of **III-4** was higher than ribavirin and resveratrol, which indicated that the introduction of heterocycle might lead to the increase of inhibitory effect. But introduction of pyridyl (**III-1**) and ortho-thienyl (**III-4**) resulted in a decline of anti-TMV activity^27^. Compound **II-5** with alkyl groups such as methyl resulted in an obvious improvement of anti-TMV activity which exhibited better curative and protection activities in vivo against TMV than ribavirin. More importantly, the inactivation activity of compound **II-5** was much higher than ribavirin. But other derivatives bearing alkyl groups **II-2**, **II-4**, **II-1**, **II-3** in series **II** showed similar or lower in vivo anti-TMV activity than ribavirin^31^. The introduction of aromatic substituents resulted in a decline of anti-TMV activity, however aromatic substituents of resveratrol with electron-donating groups such as methyl (**I-9**) or *t*-butyl (**I-10**) showed more excellent in vivo anti-TMV activity than ribavirin^27^.

### Fungicidal activity

The resveratrol and its derivatives were also evaluated for their fungicidal activities with the commercial fungicides chlorothalonil and carbendazim as the controls. Resveratrol and its derivatives all exhibited fungicidal activities to some extent against 14 kinds of plant pathogens (*Fusarium oxysporum* sp. *cucumeris; Cercospora arachidicola Hori; Physalospora piricola; Rhizoctonia cerealis; Bipolaris maydis; Colletotrichum orbiculare; Fusarium moniliforme; Alternaria solani; Fusarium graminearum; Phytophthora infestans; Phytophthora capsici; Sclerotinia sclerotiorum; Botrytis cinerea; Rhizoctonia solani*) by mycelial growth method (Table 2). As shown in Table 2, target compounds exhibited broad spectrum fungicidal activities against 14 kinds of phytopathogenic fungi at 50 μg/mL. However, compared with commercial fungicides carbendazim and chlorothalonil, these derivatives were less potent.

**Table 2:** Fungicidal activity of the compounds **I-1–I-10**, **II-1–II-5** and **III-1–III-4** against 14 kinds of phytopathogens.

Compd	Fungicidal activity %/50 μg/mL^a^													
	*F.C*^b^	*C.H*	*P.P*	*R.C*	*B.M*	*W.A*	*F.M*	*A.S*	*F.G*	*P.I*	*P.C*	*S.S*	*R.S*	*B.C*
**I-1**	18 ± 1	33 ± 1	17 ± 1	26 ± 1	5 ± 1	18 ± 2	19 ± 2	43 ± 2	36 ± 2	5 ± 1	23 ± 2	40 ± 2	12 ± 1	13 ± 1
**I-2**	14 ± 1	33 ± 1	33 ± 1	21 ± 1	5 ± 1	12 ± 2	6 ± 1	36 ± 1	36 ± 1	5 ± 1	26 ± 2	20 ± 1	16 ± 1	13 ± 1
**I-3**	18 ± 1	25 ± 1	50 ± 1	33 ± 1	27 ± 1	35 ± 2	31 ± 1	43 ± 2	21 ± 1	41 ± 2	23 ± 1	20 ± 1	48 ± 2	7 ± 2
**I-4**	5 ± 1	33 ± 1	33 ± 1	5 ± 1	14 ± 2	6 ± 1	13 ± 2	21 ± 1	14 ± 1	23 ± 2	29 ± 1	20 ± 1	15 ± 3	13 ± 1
**I-5**	9 ± 1	33 ± 1	50 ± 1	37 ± 3	18 ± 1	6 ± 1	13 ± 1	21 ± 1	27 ± 2	27 ± 1	26 ± 2	20 ± 1	31 ± 1	27 ± 3
**I-6**	5 ± 1	8 ± 1	17 ± 2	16 ± 3	9 ± 1	6 ± 1	19 ± 3	21 ± 1	36 ± 2	5 ± 1	16 ± 1	30 ± 1	16 ± 1	33 ± 1
**I-7**	5 ± 1	33 ± 1	17 ± 2	21 ± 1	14 ± 2	12 ± 3	13 ± 2	14 ± 1	14 ± 1	9 ± 1	16 ± 1	10 ± 1	25 ± 2	13 ± 1
**I-8**	14 ± 2	25 ± 1	8 ± 1	11 ± 2	14 ± 2	6 ± 1	13 ± 1	14 ± 1	36 ± 2	9 ± 1	16 ± 1	20 ± 1	20 ± 2	20 ± 1
**I-9**	5 ± 1	17 ± 2	33 ± 1	16 ± 3	18 ± 1	12 ± 3	6 ± 1	7 ± 1	36 ± 2	14 ± 2	16 ± 1	30 ± 1	5 ± 2	13 ± 1
**I-10**	5 ± 1	33 ± 1	25 ± 1	21 ± 1	14 ± 2	12 ± 3	13 ± 2	14 ± 1	21 ± 1	14 ± 2	16 ± 1	20 ± 1	15 ± 3	7 ± 2
**II-1**	5 ± 1	33 ± 1	42 ± 3	37 ± 3	17 ± 2	6 ± 3	19 ± 2	7 ± 1	36 ± 2	14 ± 2	19 ± 1	30 ± 1	31 ± 1	33 ± 1
**II-2**	14 ± 2	8 ± 1	17 ± 2	16 ± 3	5 ± 1	6 ± 3	13 ± 2	7 ± 1	21 ± 1	14 ± 2	19 ± 1	30 ± 1	20 ± 3	20 ± 1
**II-3**	18 ± 1	8 ± 1	42 ± 3	21 ± 1	5 ± 1	6 ± 3	19 ± 3	7 ± 1	7 ± 1	5 ± 2	19 ± 1	20 ± 1	31 ± 1	33 ± 1
**II-4**	18 ± 1	17 ± 2	33 ± 1	32 ± 2	18 ± 1	18 ± 2	25 ± 1	14 ± 1	7 ± 1	5 ± 2	26 ± 3	20 ± 1	21 ± 1	20 ± 1
**II-5**	5 ± 2	33 ± 1	42 ± 2	42 ± 1	18 ± 1	47 ± 1	13 ± 1	36 ± 2	36 ± 2	18 ± 1	29 ± 1	10 ± 1	36 ± 1	13 ± 1
**III-1**	14 ± 2	25 ± 1	8 ± 1	26 ± 3	14 ± 2	6 ± 3	25 ± 1	7 ± 1	7 ± 1	18 ± 1	13 ± 3	20 ± 1	31 ± 1	20 ± 1
**III-2**	14 ± 2	8 ± 1	8 ± 1	21 ± 1	5 ± 1	6 ± 3	13 ± 2	14 ± 1	21 ± 1	14 ± 2	23 ± 2	10 ± 1	15 ± 3	7 ± 2
**III-3**	5 ± 1	17 ± 2	8 ± 1	21 ± 1	14 ± 2	18 ± 2	6 ± 1	21 ± 1	36 ± 2	14 ± 2	23 ± 2	20 ± 1	16 ± 1	20 ± 1
**III-4**	14 ± 2	8 ± 1	33 ± 1	11 ± 1	5 ± 1	17 ± 2	6 ± 1	36 ± 2	36 ± 2	9 ± 1	19 ± 1	20 ± 1	12 ± 1	20 ± 1
Resveratr-ol^d^	27 ± 1	42 ± 2	25 ± 1	42 ± 1	36 ± 1	35 ± 1	13 ± 1	43 ± 2	21 ± 1	36 ± 1	42 ± 2	40 ± 1	48 ± 1	40 ± 1
Chlorothal-onil^c^	100	70 ± 1	100	75 ± 1	< 50	100	< 50	100	100	92 ± 1	92 ± 1	87 ± 1	100	100
Carbenda-zim^c^	< 50	< 50	< 50	< 50	100	< 50	100	< 50	100	100	100	100	100	< 50

^a^Average of three replicates. All results are expressed as the mean ± SD.

^b^F.C, *Fusarium oxysporium f. sp. cucumeris*; C.H, *Cercospora arachidicola Hori*; P.P, *Physalospora piricola*; R.C, *Rhizoctonia cerealis*; B.M, *Bipolaris maydis*; W.A, *Watermelon anthracnose*; F.M, *Fusarium moniliforme*; A.S, *Alternaria solani*; F.G, *Fusarium graminearum*; P.I, *Phytophthora infestans*; P.C, *Phytophthora capsici*; S.S, *Sclerotinia sclerotiorum*; R.S, *Rhizoctonia solani*; B.C, *Botrytis cinerea.*

^c^The commercial agricultural fungicides chlorothalonil and carbendazim were used for comparison of antifungal activities.

^d^Positive control.

### Insecticidal activities

The insecticidal activities of target compounds and resveratrol against oriental armyworm (*Mythimna separata*), cotton bollworm (*Helicoverpa armigera*), corn borer (*Ostrinia furnacalis*), and Mosquito (*Culex pipiens pallens*) are listed in Table 3. The results indicated that many compounds exhibited higher activities than resveratrol. Especially compounds **II-3**, **III-3**, and **III-4** exhibited obviously higher activities against oriental armyworm (70%, 70%, 60% at 600 μg/mL) than resveratrol.

**Table 3:** Insecticidal activities of the target compounds **I-1–I-10**, **II-1–II-5** and **III-1–III-4** (mortality^a^, percent).

Compd	Cotton bollworm (600 μg/mL)	Corn borer (600 μg/mL)	Oriental armyworm (600 μg/mL)	Mosquito (10 μg/mL)
**I-1**	20 ± 2	5 ± 1	20 ± 2	5 ± 1
**I-2**	0	0	10 ± 2	0
**I-3**	5 ± 1	0	10 ± 2	0
**I-4**	0	0	5 ± 1	20 ± 2
**I-5**	0	0	0	0
**I-6**	10 ± 2	10 ± 2	15 ± 2	5 ± 1
**I-7**	0	5 ± 1	10 ± 2	0
**I-8**	10 ± 2	15 ± 2	20 ± 2	0
**I-9**	0	0	0	5 ± 1
**I-10**	20 ± 2	10 ± 2	40 ± 2	0
**II-1**	5 ± 1	20 ± 2	30 ± 2	10 ± 2
**II-2**	0	15 ± 2	20 ± 2	0
**II-3**	30 ± 2	35 ± 2	70 ± 3	5 ± 1
**II-4**	10 ± 2	10 ± 2	30 ± 2	20 ± 2
**II-5**	15 ± 2	20 ± 2	50 ± 2	10 ± 2
**III-1**	5 ± 1	5 ± 1	10 ± 1	20 ± 2
**III-2**	10 ± 2	15 ± 2	40 ± 2	0
**III-3**	25 ± 2	35 ± 2	70 ± 2	30 ± 2
**III-4**	30 ± 2	25 ± 2	60 ± 2	0
Resveratrol^b^	5 ± 1	0	10 ± 2	70 ± 2

^a^Average of three replicates. All results are expressed as the mean ± standard deviation (SD).

^b^Positive control.

However, though some compounds exhibited insecticidal activity on some species to some extent, the potency of these compounds as insecticide was not comparable with that of commercial insecticides. More modification on the structure should be conducted.

## Conclusion

In summary, the natural product resveratrol provides an unparalleled source of inspiration for the screening of antiviral drugs. With resveratrol as lead compound by chemical modification, a series of resveratrol ester derivatives were designed and synthesized. Their activities against TMV were evaluated. The optimum compounds **III-2**, **III-4**, and **II-5**, **I-9**, **I-10** displayed higher activity than commercial plant virucide ribavirin. This paper also discussed the anti-TMV activities and the structure–activity relationships of resveratrol ester derivatives, providing a reference for the development of new drugs. Resveratrol as important phytoalexin, its structure is simple and is a kind of lead compound which is extremely potential in the field of pesticide development, so the structure modification of resveratrol is a very valuable and meaningful research work.

## Supplementary Information

Supplementary Information.
