# Supplementary material for: Natural phytoalexin stilbene compound resveratrol and its derivatives as anti-tobacco mosaic virus and anti-phytopathogenic fungus agents
Source: Sci Rep. 2021 Aug 13;11:16509. doi: 10.1038/s41598-021-96069-1 (PMC8363727; doi:10.1038/s41598-021-96069-1)
Supplement: Supplementary file 1 — Supplementary Information. [file 41598_2021_96069_MOESM1_ESM.docx]

*Supporting Information*

**Natural phytoalexin stilbene compound resveratrol and its derivatives as anti-tobacco mosaic virus and anti-phytopathogenic fungus agents**

**Pengfei Song^1^, Xiuling Yu^*1^, Wenqiang Yang^1^, Qingmin Wang^*2, 3^**

*^1^College of Pharmacy, Linyi University, Linyi 276000, People’s Republic of China.*

*^2^State Key Laboratory of Elemento-Organic Chemistry, Research Institute of Elemento-Organic Chemistry, College of Chemistry, Nankai University, Tianjin 300071, People’s Republic of China.*

*^3^Collaborative Innovation Center of Chemical Science and Engineering (Tianjin), Tianjin 300071, People’s Republic of China.*

* Corresponding author: [yxlwell@163.com](mailto:yxlwell@163.com) (X.-L. Yu), [wangqm@nankai.edu.cn](mailto:wangqm@nankai.edu.cn) (Q.-M. Wang).

**Fig. 1** The design and synthesis of compounds **I-1−I-10, II-1−II-5** and **III-1−III-4**.

 **Fig. 2** Chemical structures of **I-1−I-10**.

**Fig. 3** Chemical structures of **II-1−II-5**.

 **Fig. 4** Chemical structures of **III-1−III-4**.

**^1^H NMR spectrum and ^13^C NMR spectrum of target compounds**


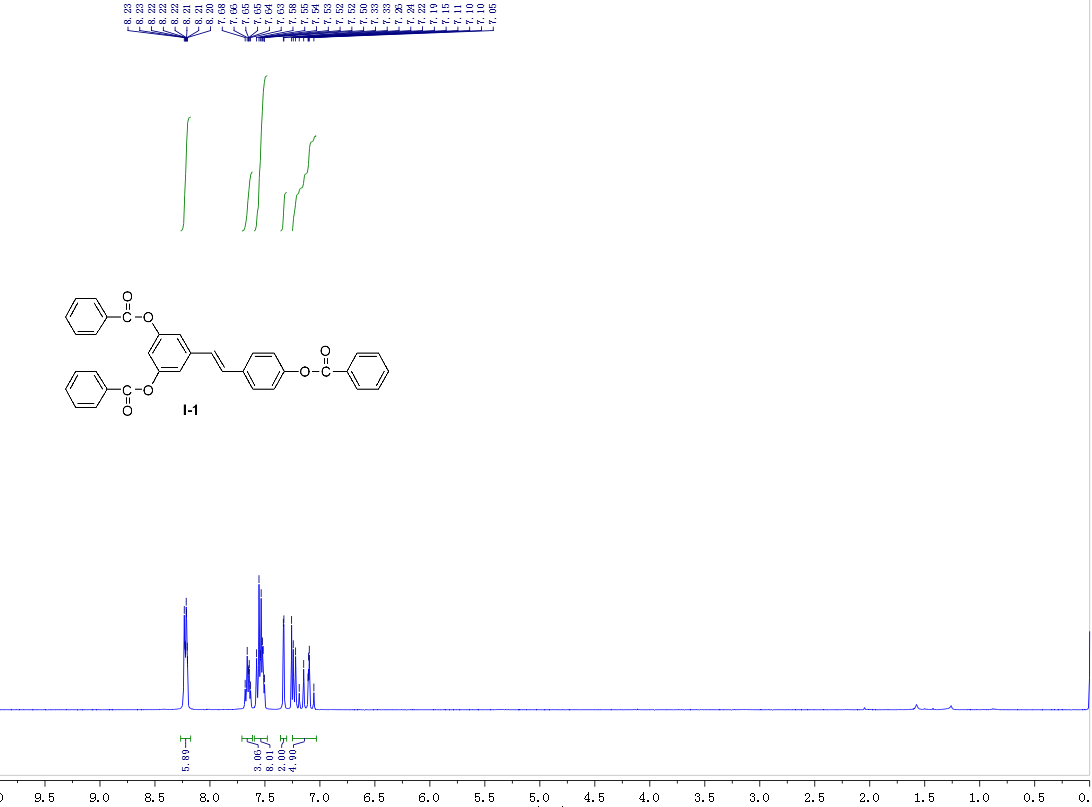


**Fig. 5** ^1^H NMR spectrum of **I-1**


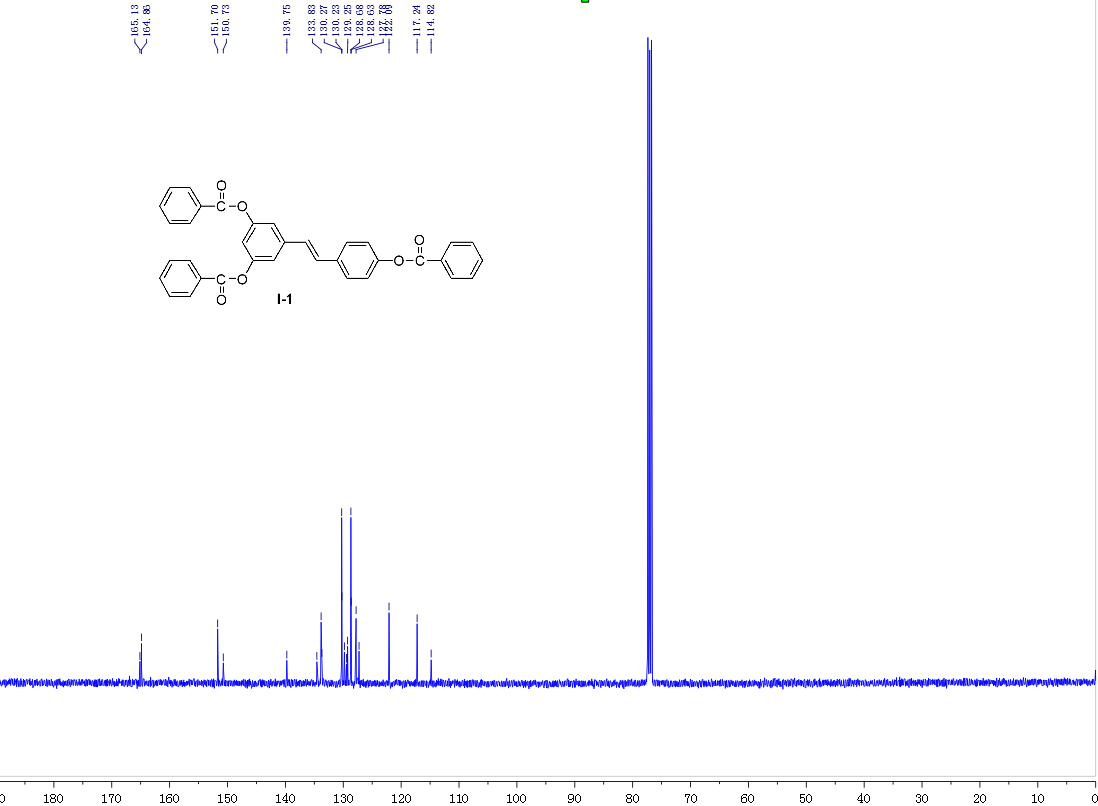


**Fig. 6** ^13^C NMR spectrum of **I-1**


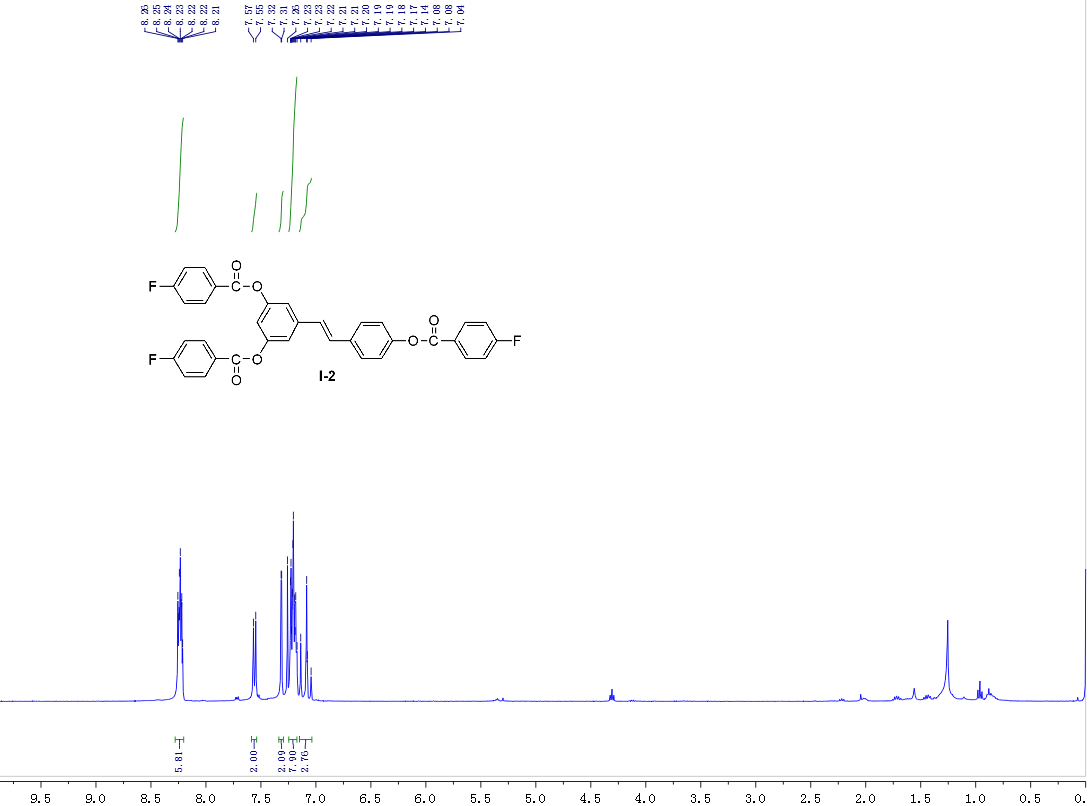


**Fig. 7** ^1^H NMR spectrum of **I-2**


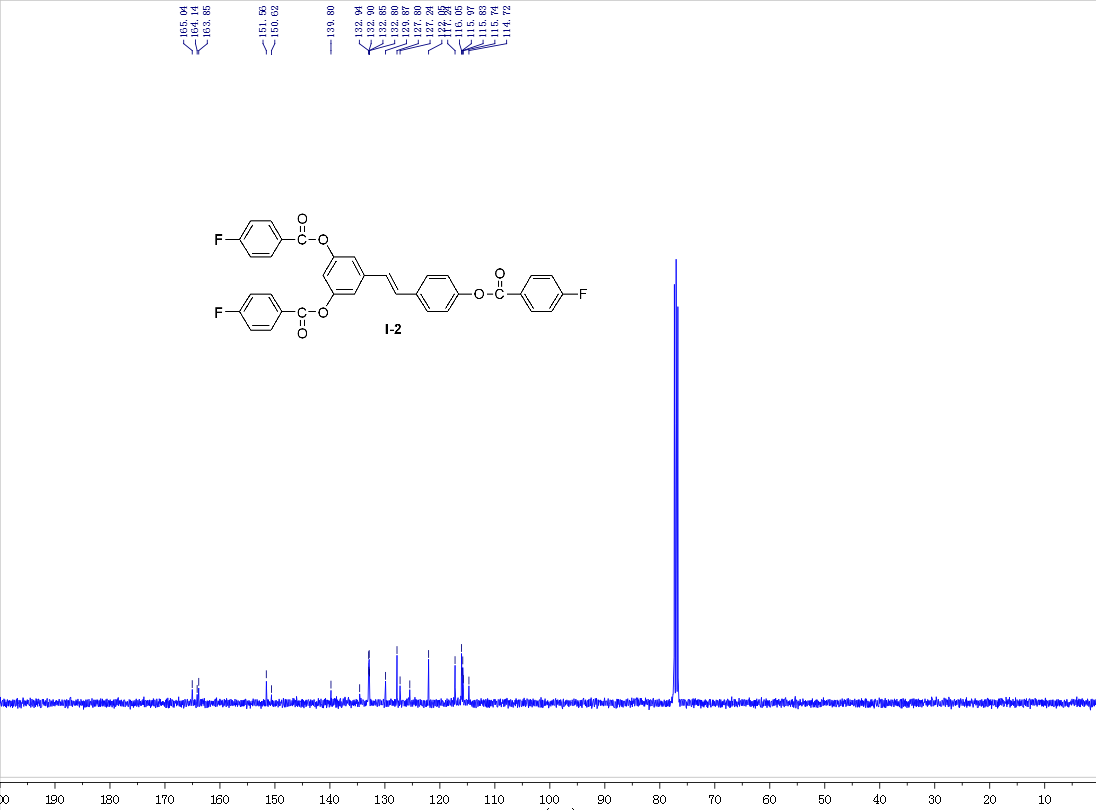


**Fig. 8** ^13^C NMR spectrum of **I-2**


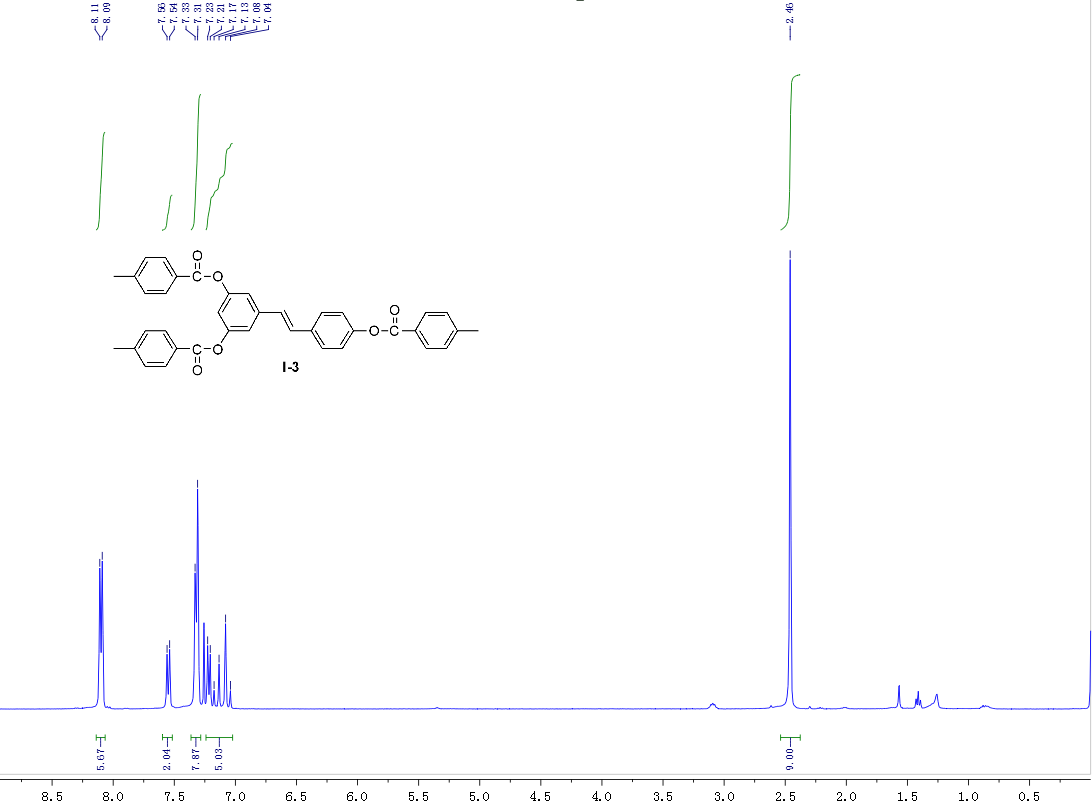


**Fig. 9** ^1^H NMR spectrum of **I-3**


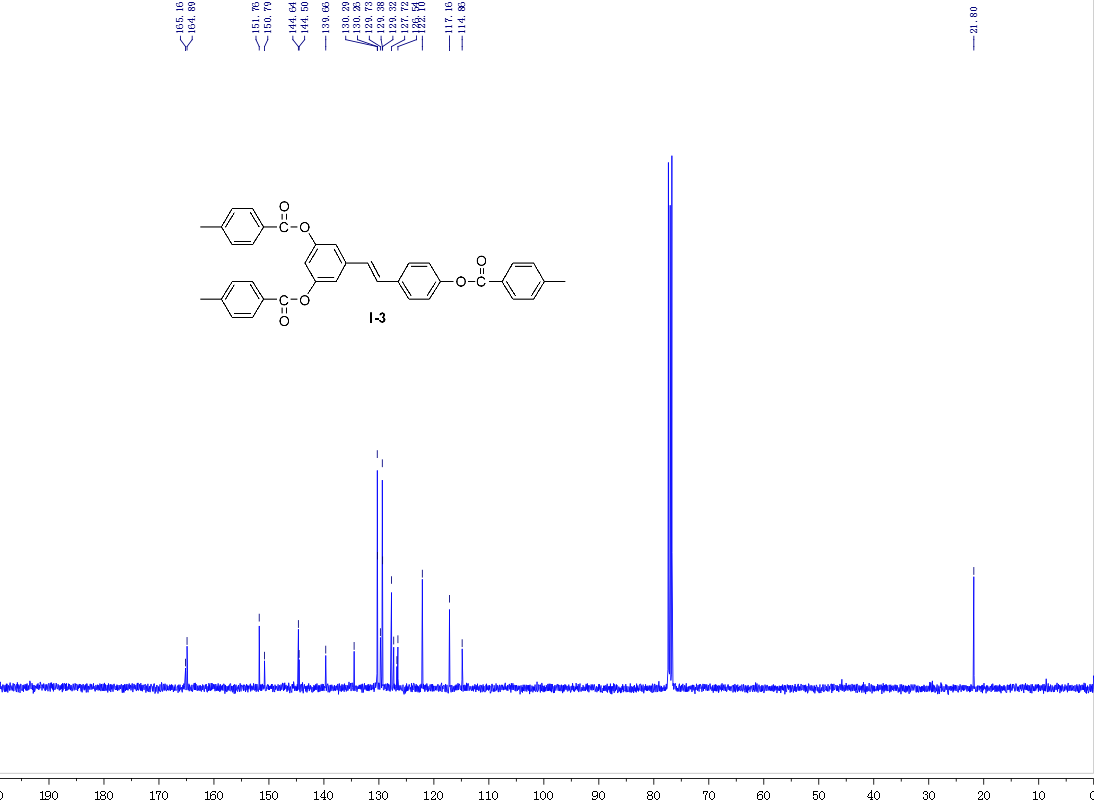


**Fig. 10** ^13^C NMR spectrum of **I-3**


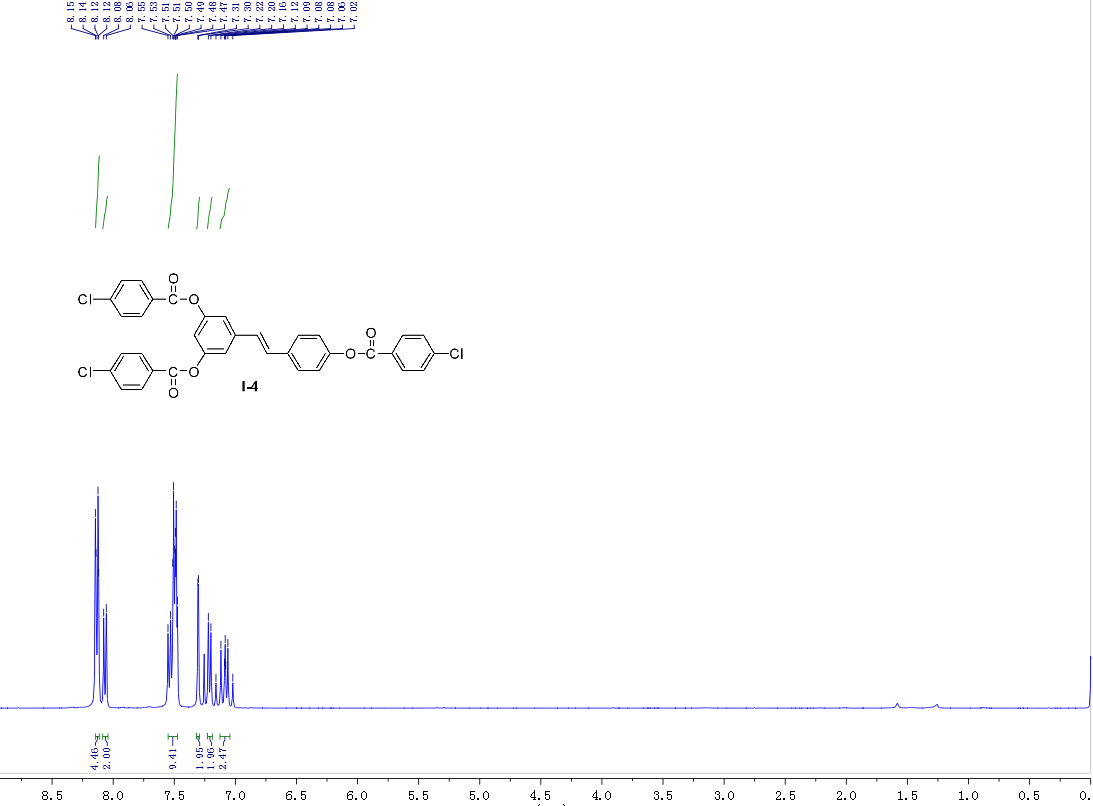


**Fig. 11** ^1^H NMR spectrum of **I-4**


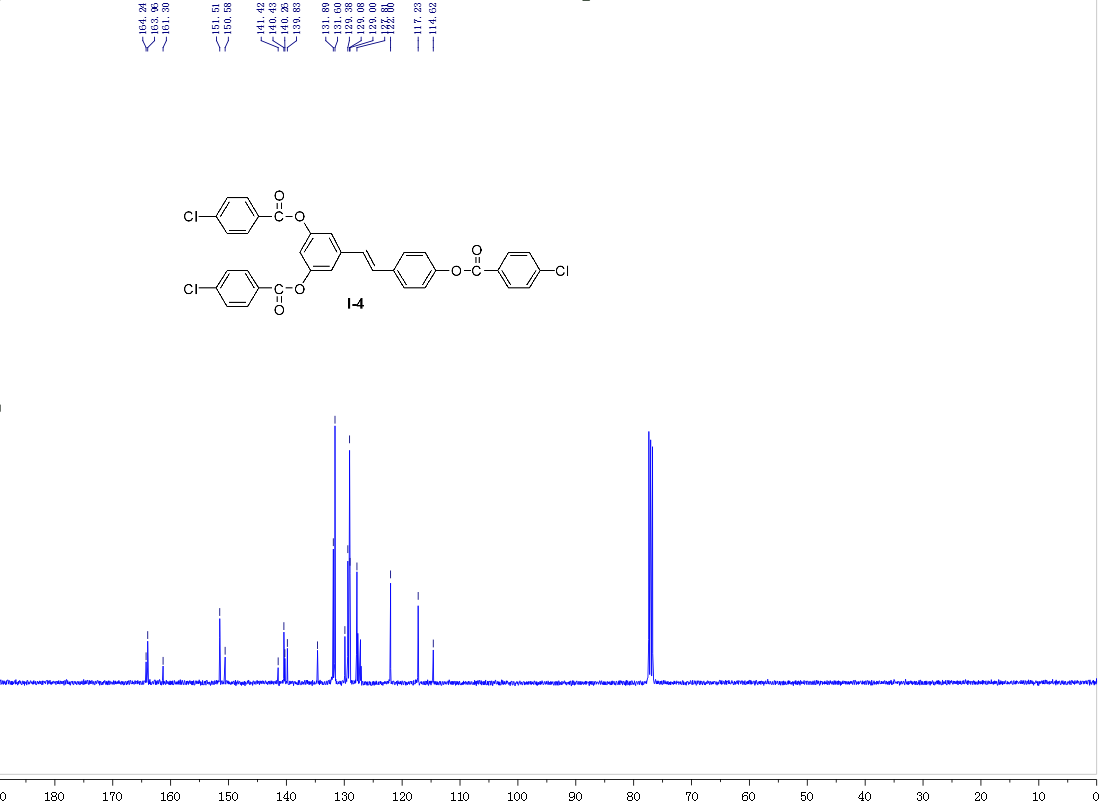


**Fig. 12** ^13^C NMR spectrum of **I-4**


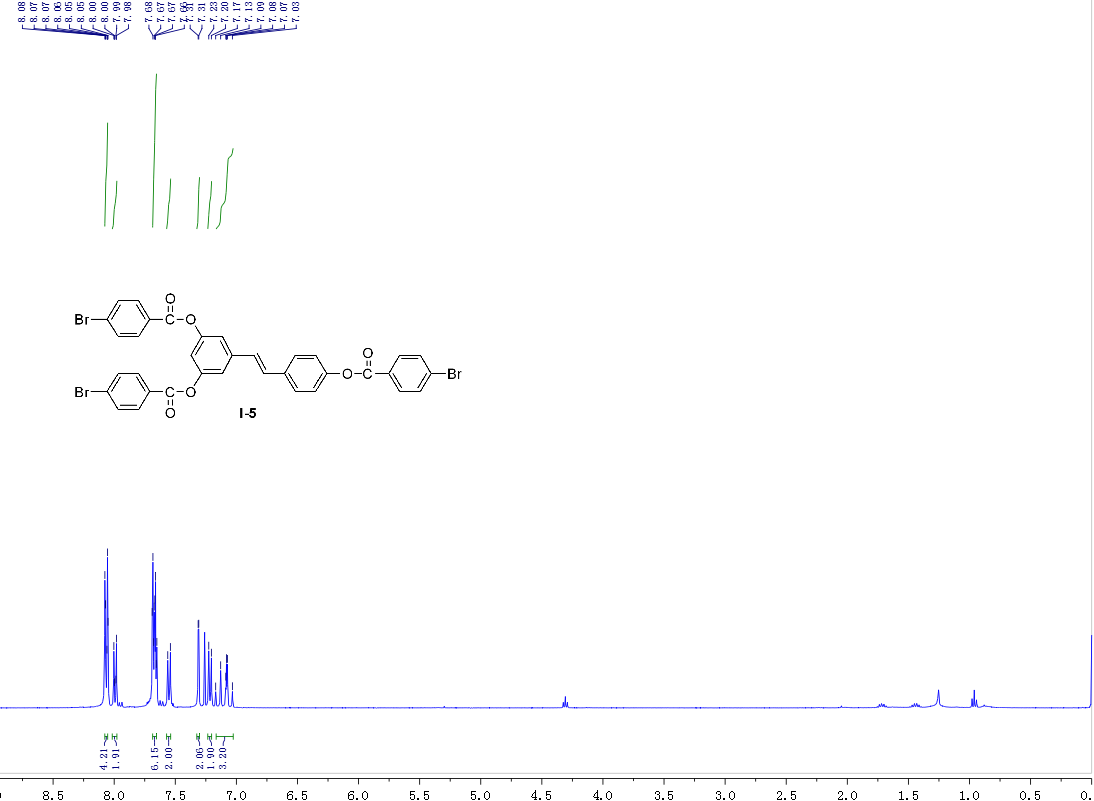


**Fig. 13** ^1^H NMR spectrum of **I-5**


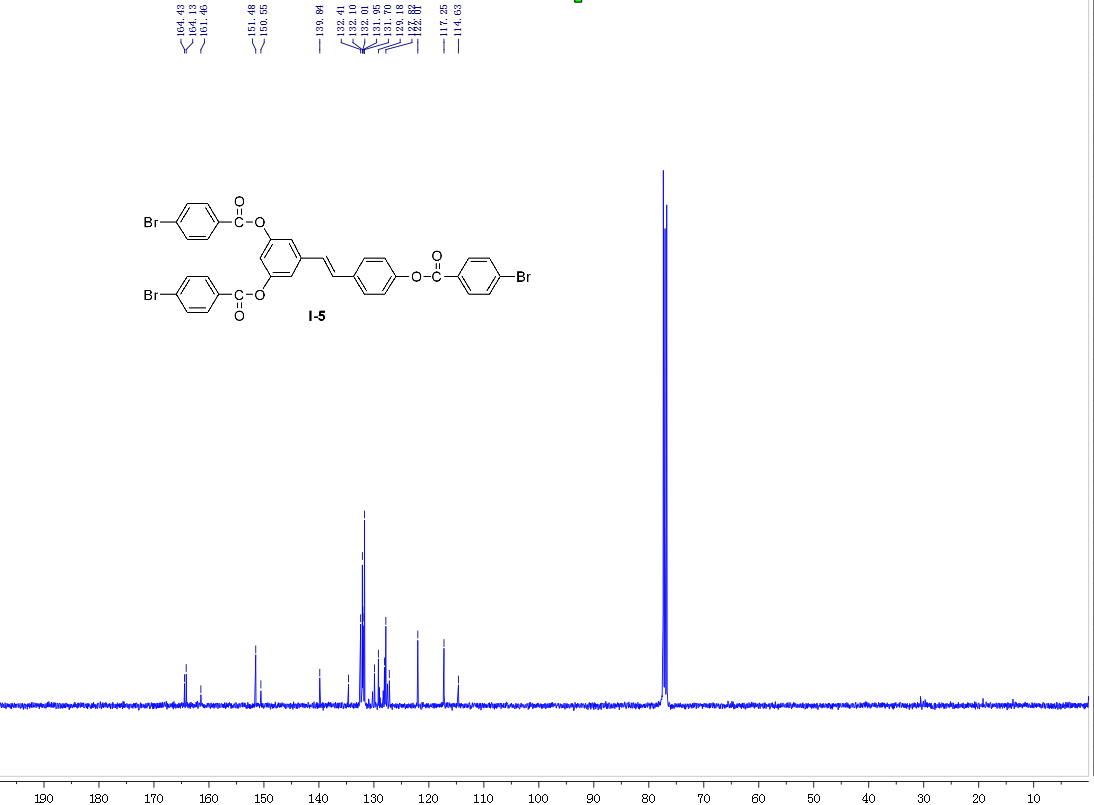


**Fig. 14** ^13^C NMR spectrum of **I-5**


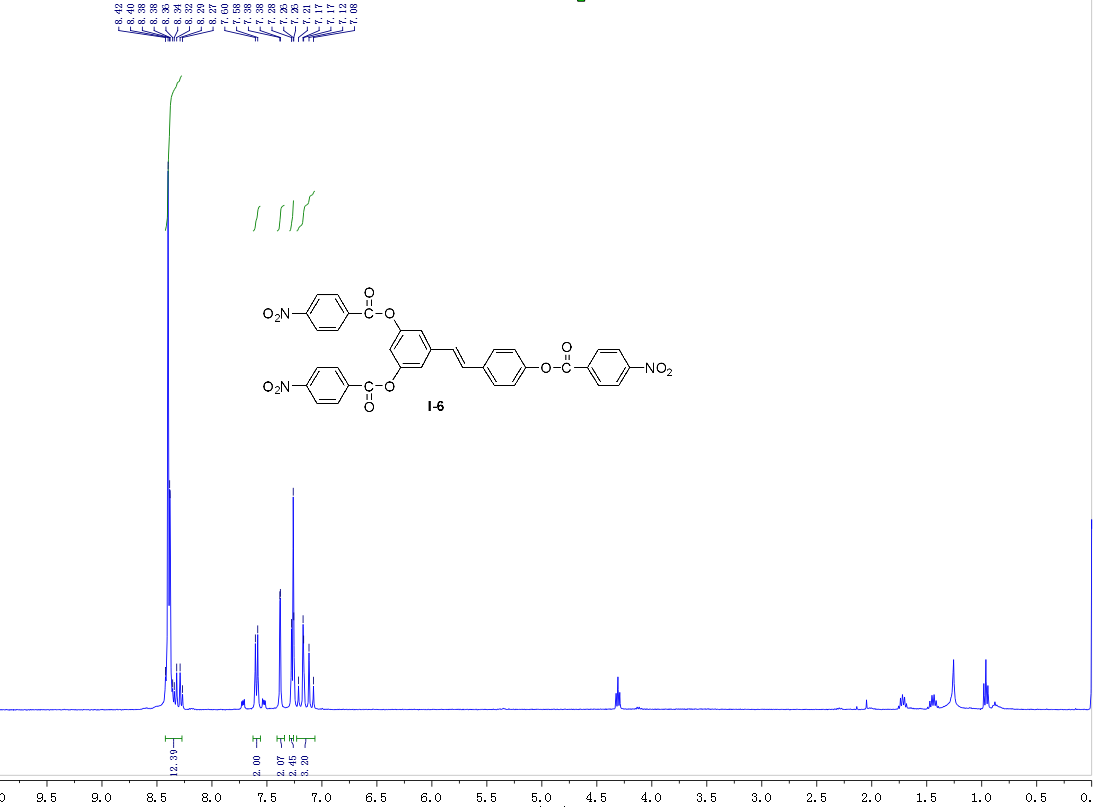


**Fig. 15** ^1^H NMR spectrum of **I-6**


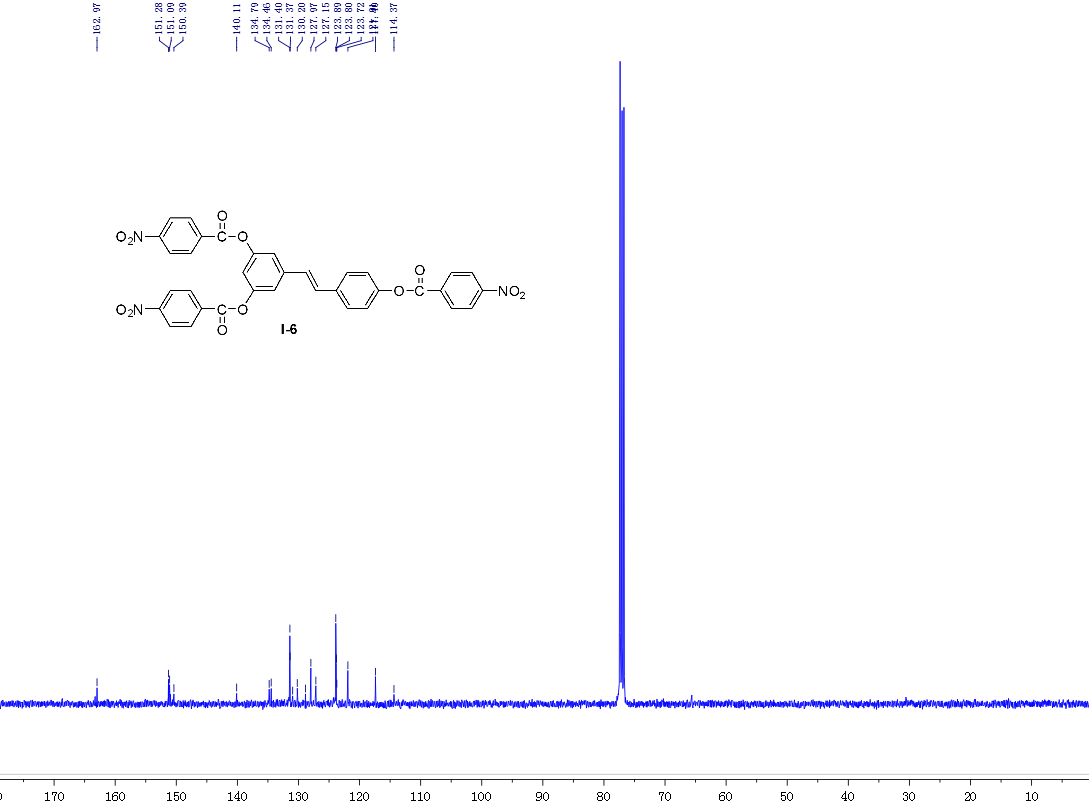


**Fig. 16** ^13^C NMR spectrum of **I-6**


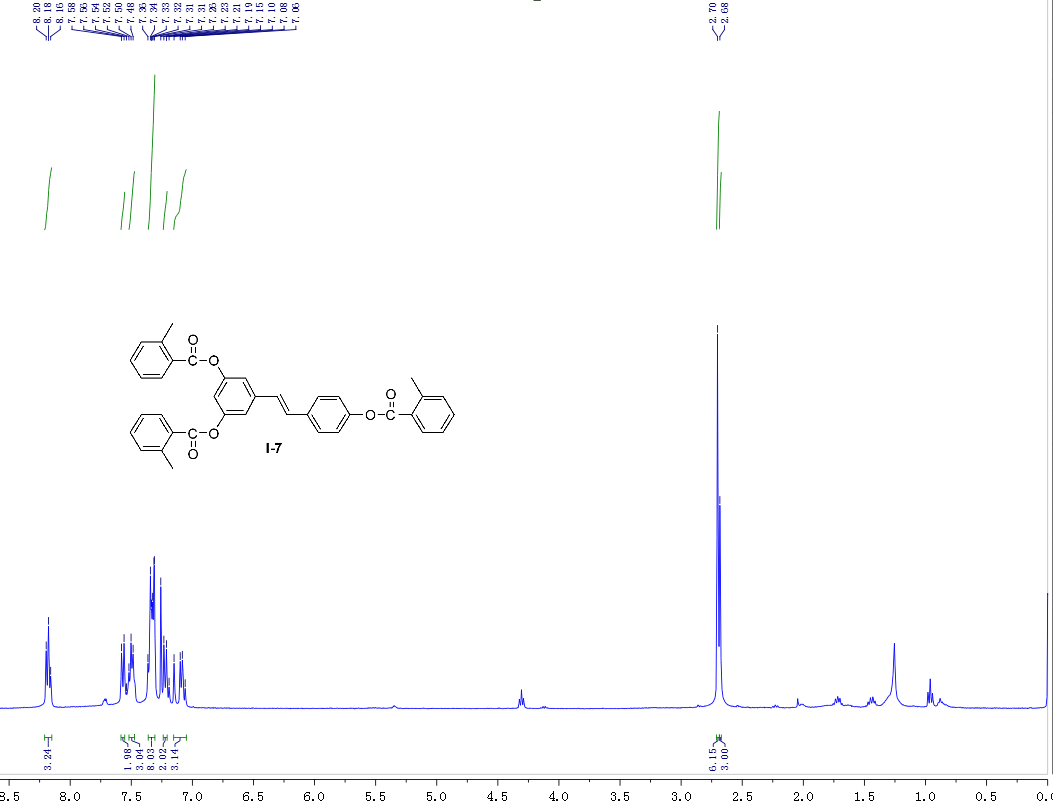


**Fig. 17** ^1^H NMR spectrum of **I-7**


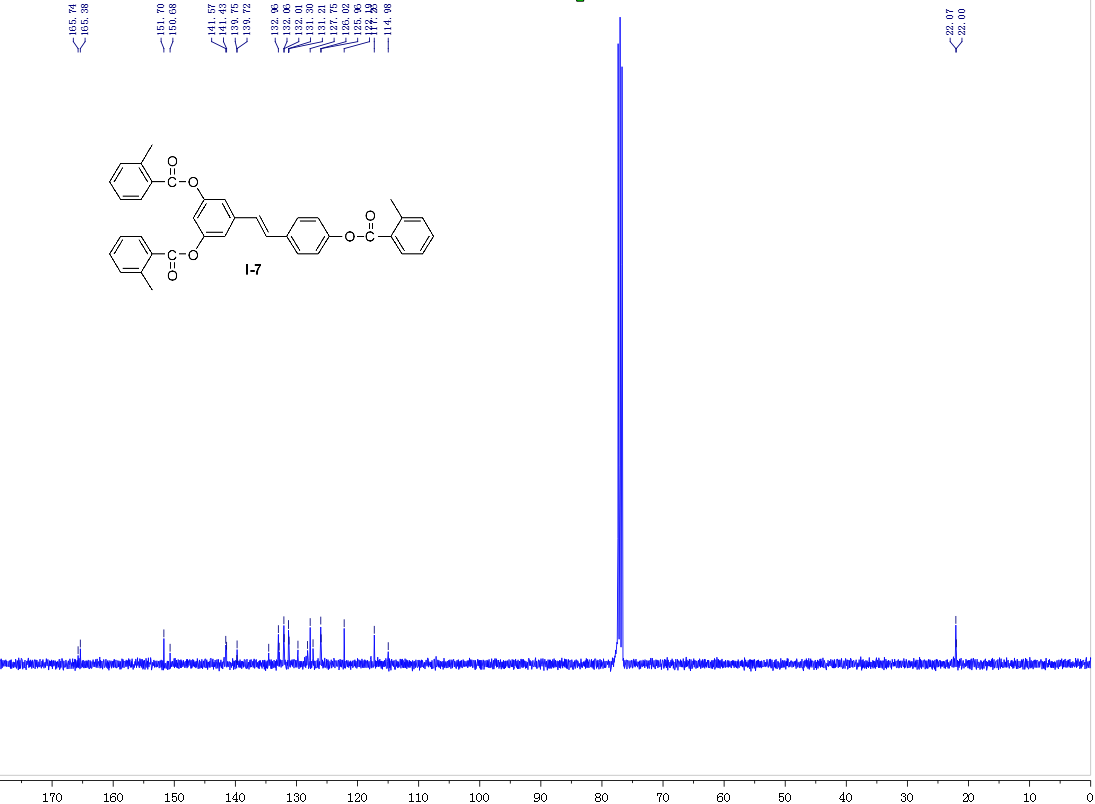


**Fig. 18** ^13^C NMR spectrum of **I-7**


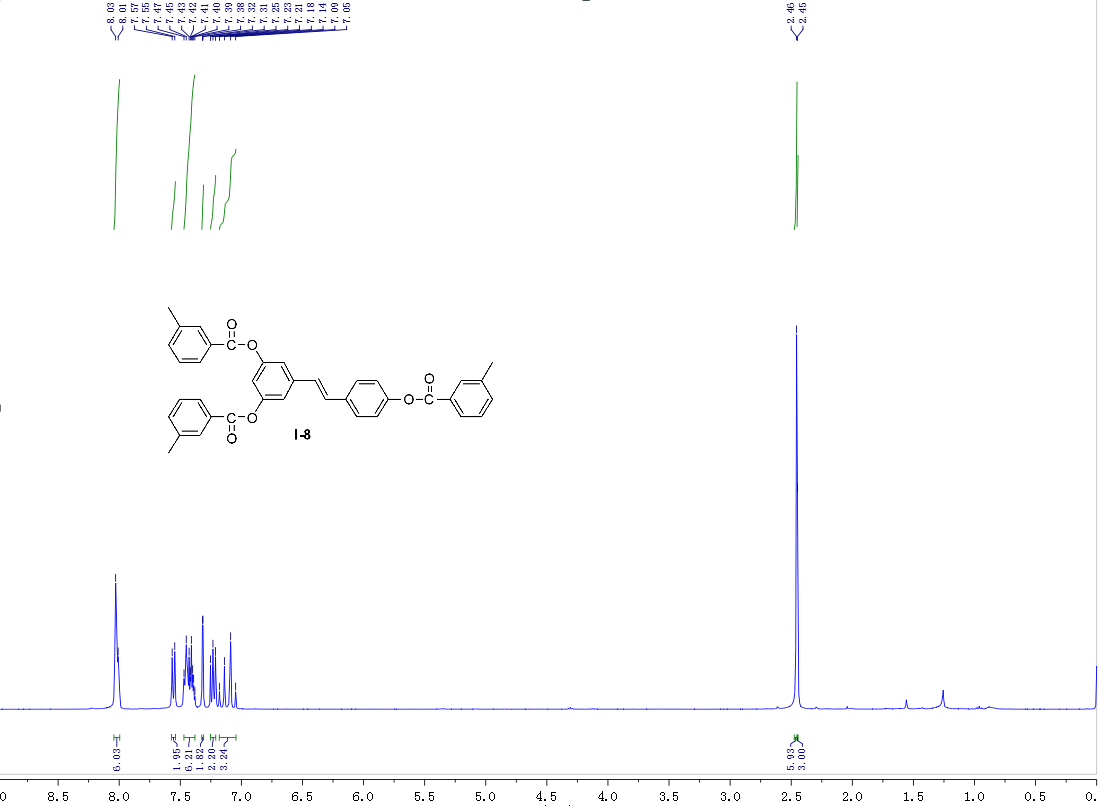


**Fig. 19** ^1^H NMR spectrum of **I-8**


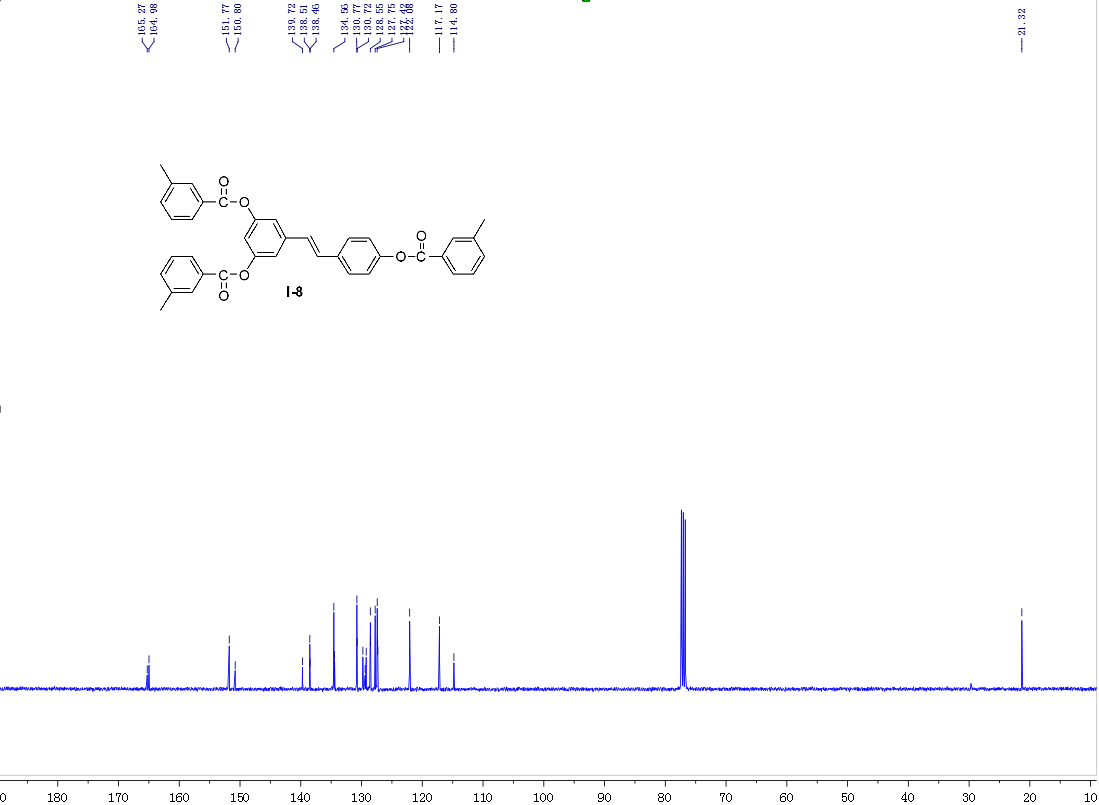


**Fig. 20** ^13^C NMR spectrum of **I-8**


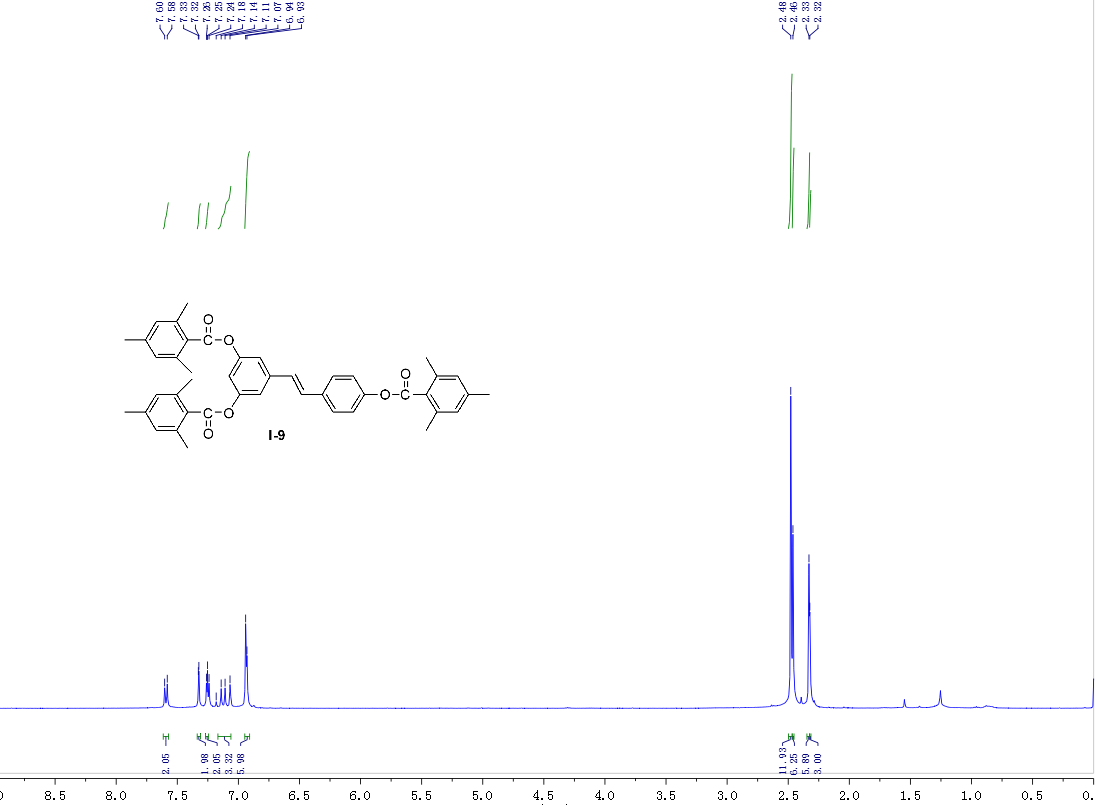


**Fig. 21** ^1^H NMR spectrum of **I-9**


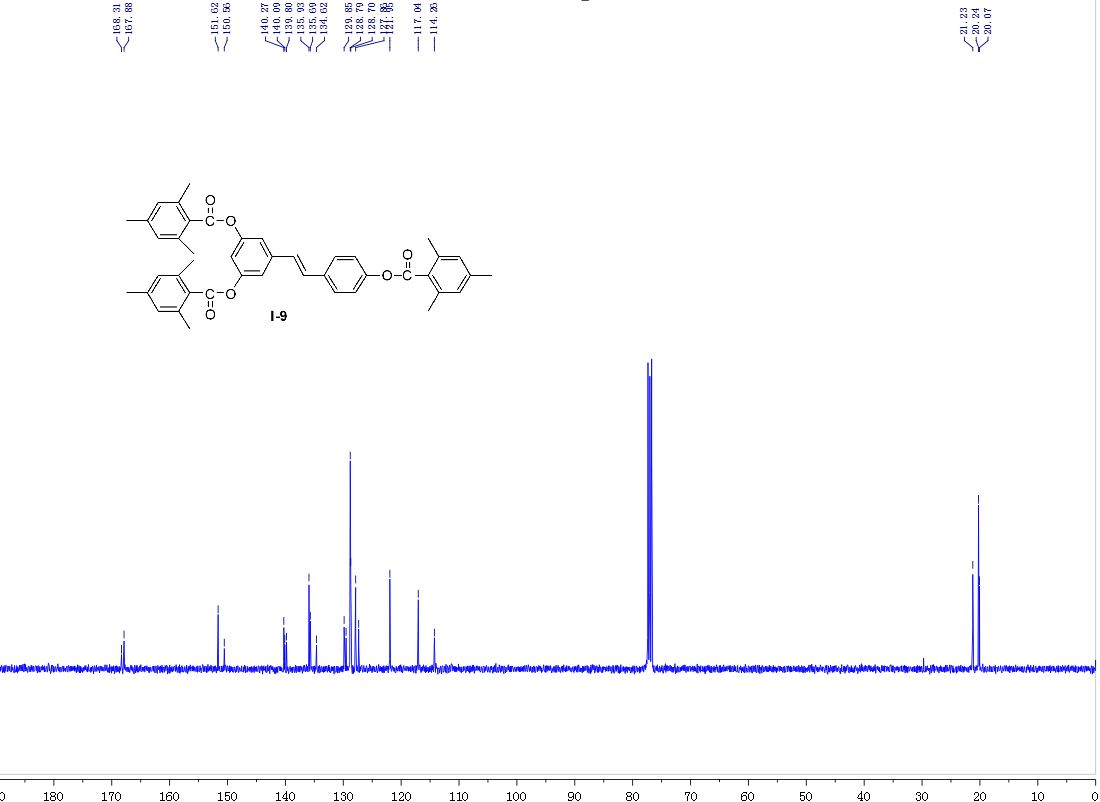


**Fig. 22** ^13^C NMR spectrum of **I-9**


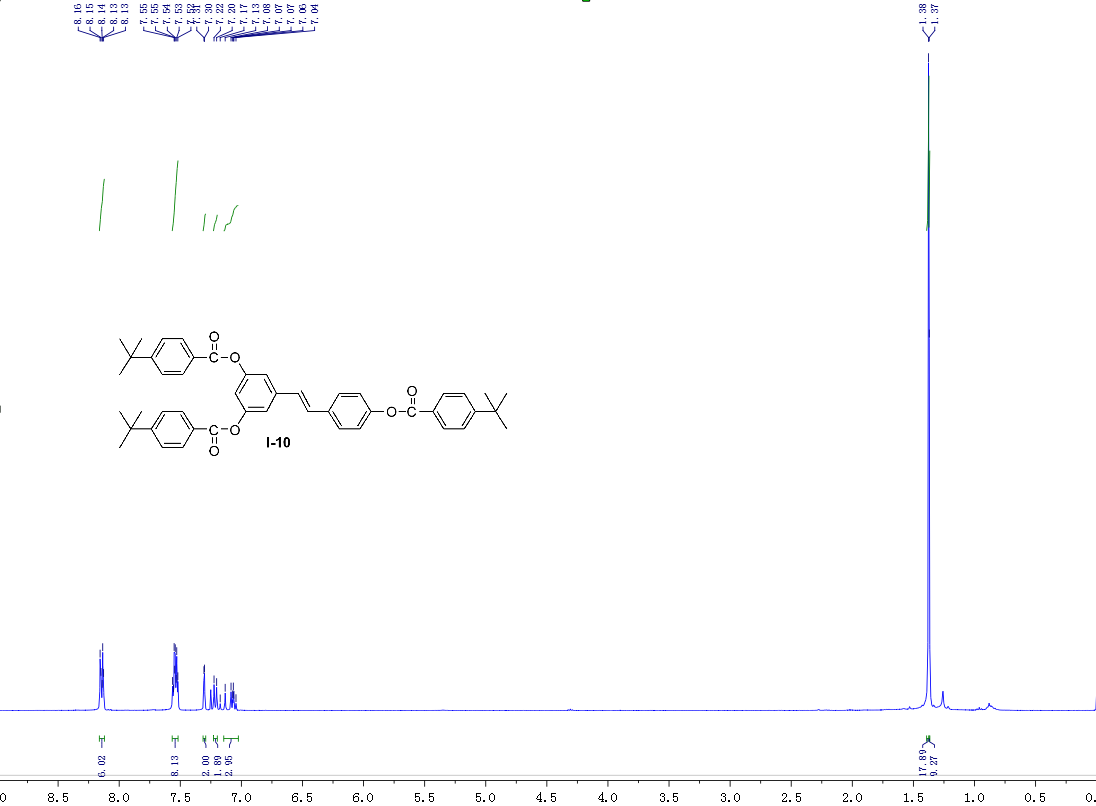


**Fig. 23** ^1^H NMR spectrum of **I-10**


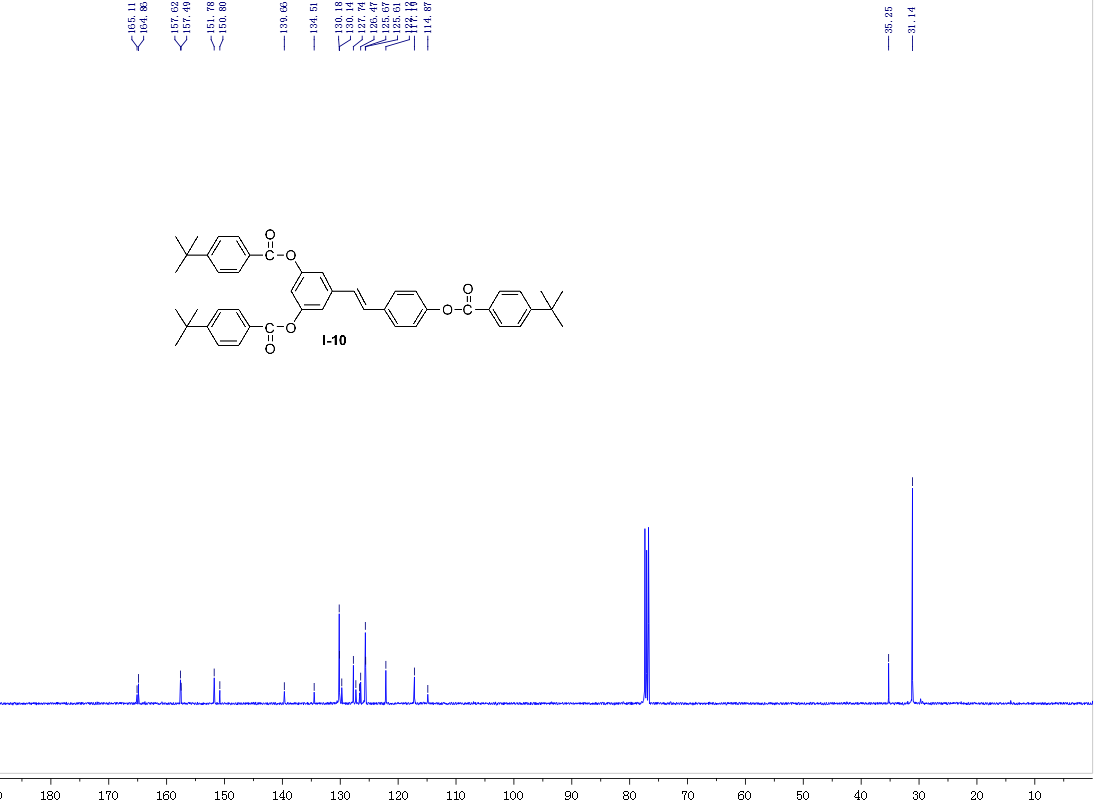


**Fig. 24** ^13^C NMR spectrum of **I-10**


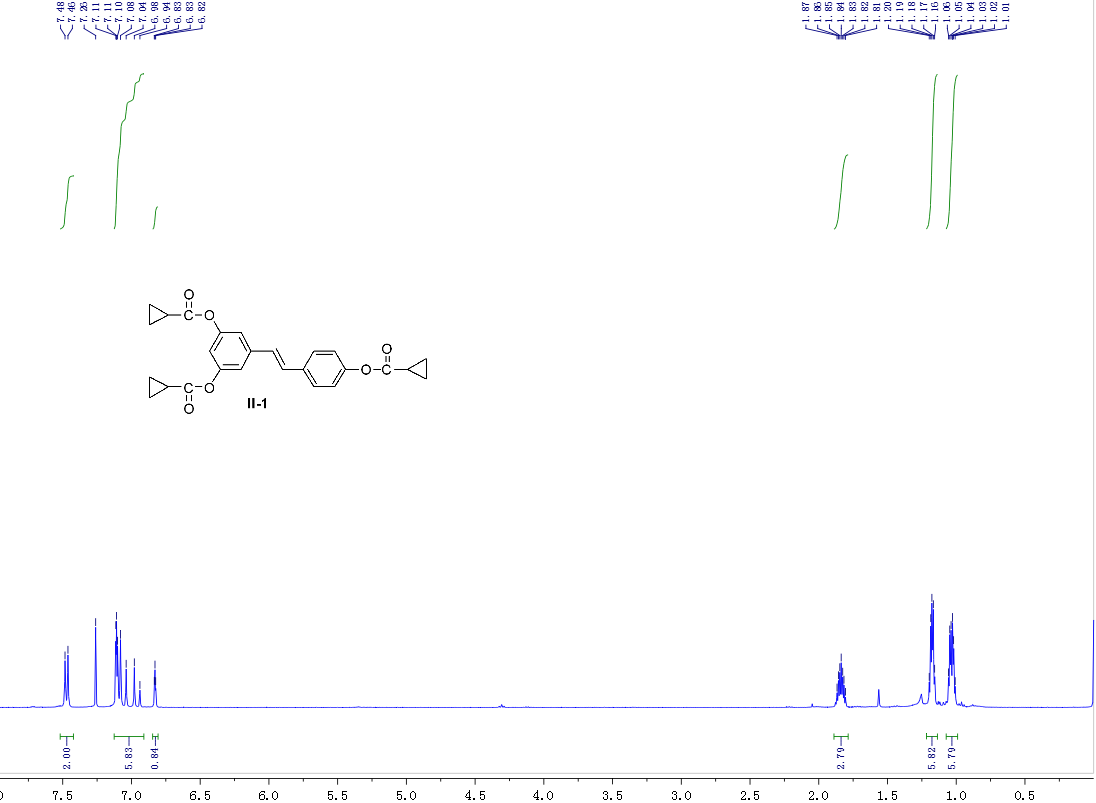


**Fig. 25** ^1^H NMR spectrum of **II-1**


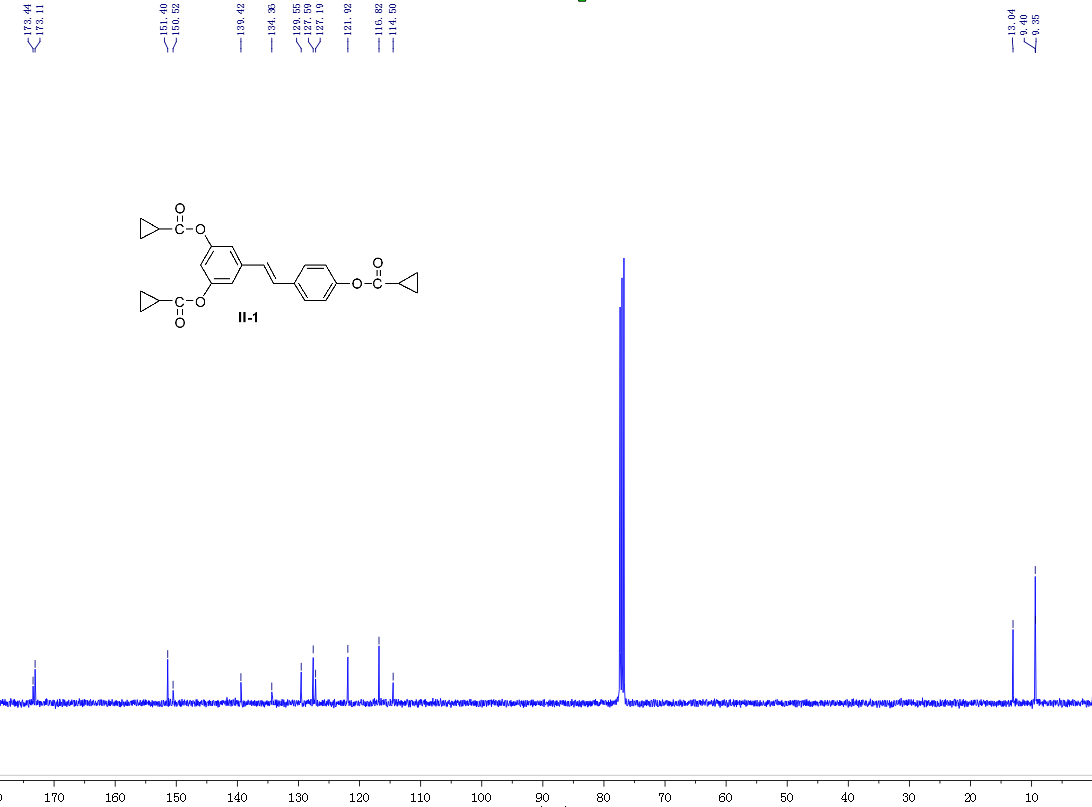


**Fig. 26** ^13^C NMR spectrum of **II-1**


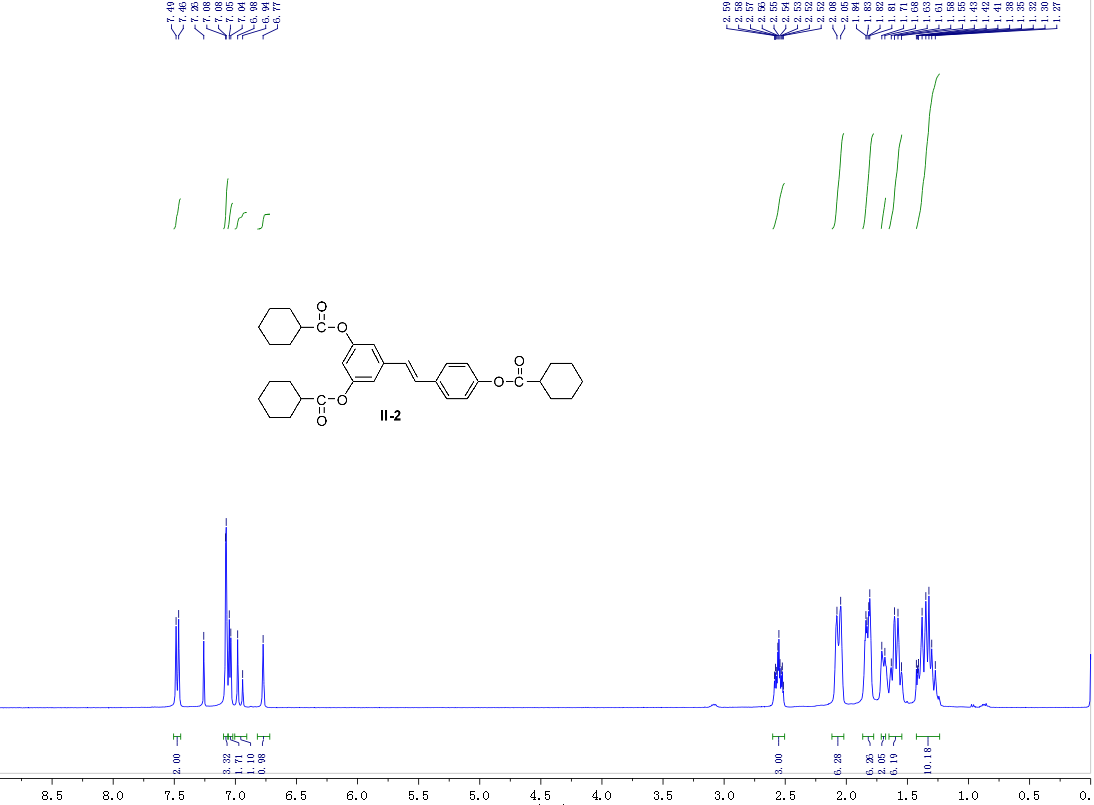


**Fig. 27** ^1^H NMR spectrum of **II-2**


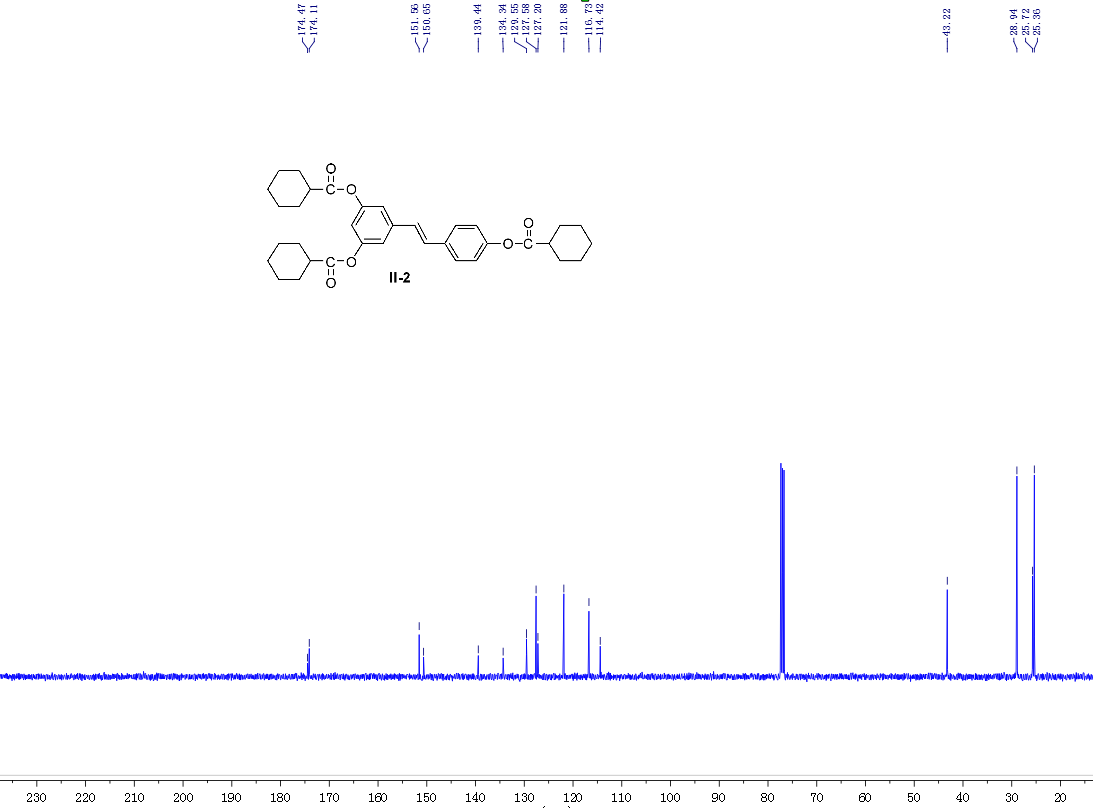


**Fig. 28** ^13^C NMR spectrum of **II-2**


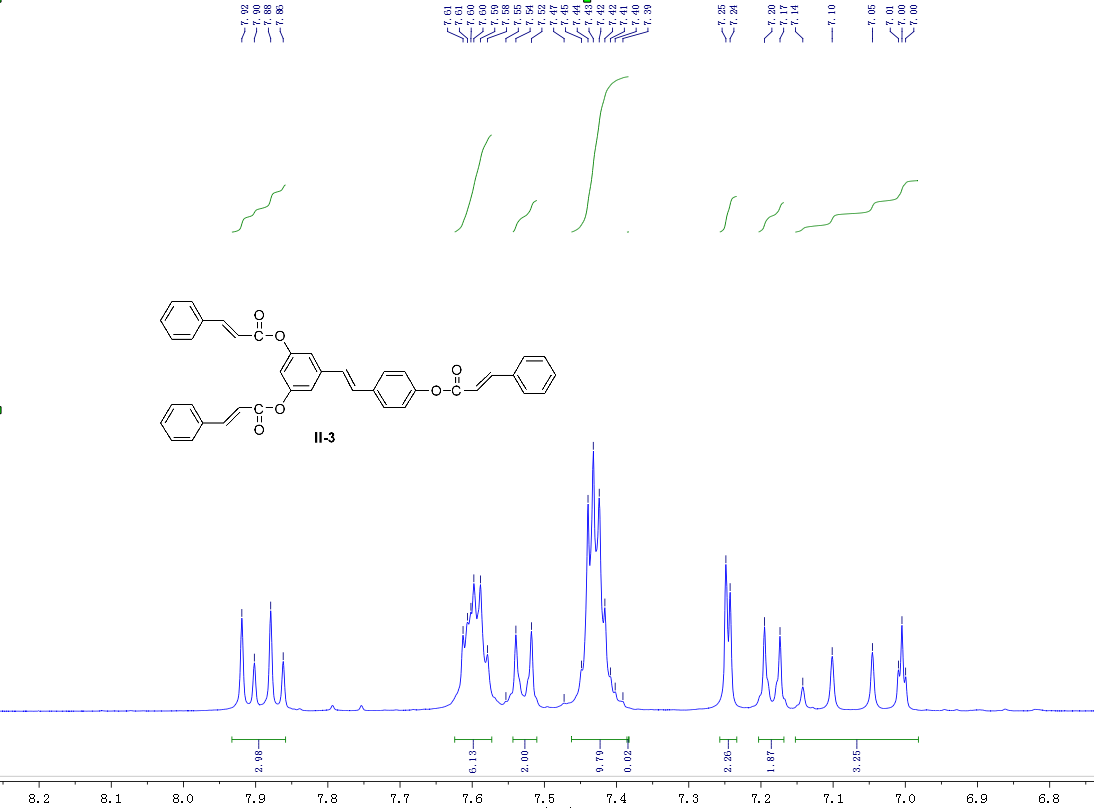


**Fig. 29** ^1^H NMR spectrum of **II-3**


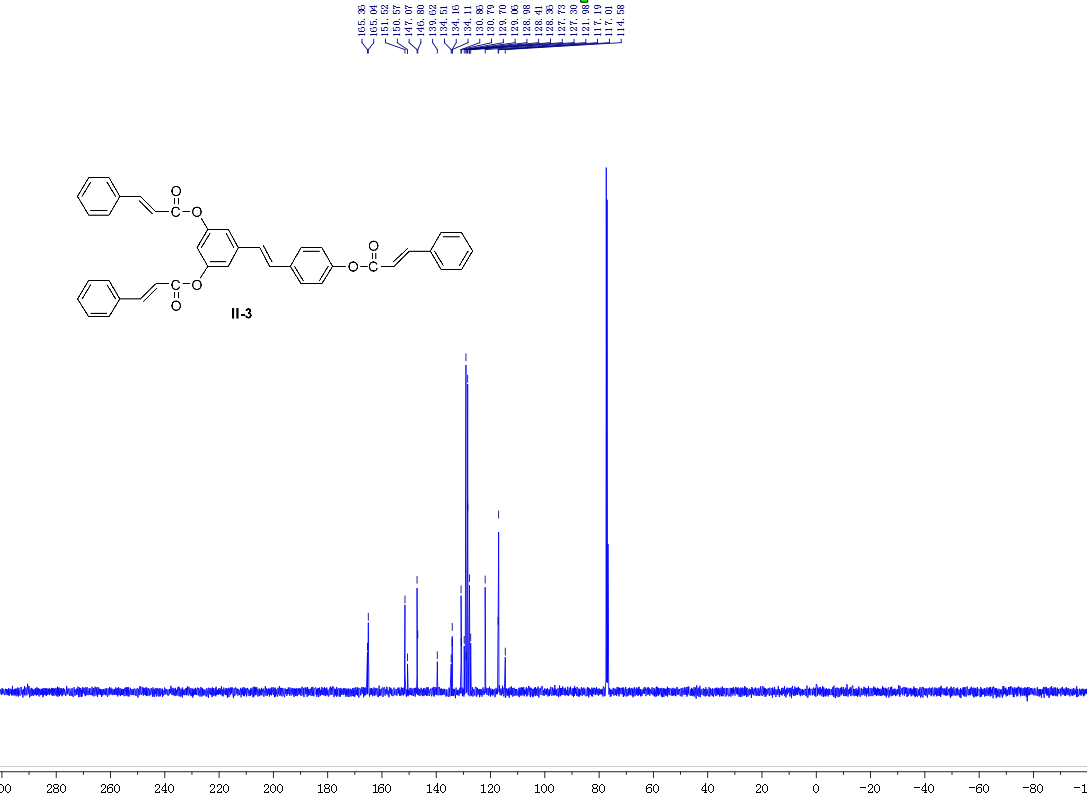


**Fig. 30** ^13^C NMR spectrum of **II-3**


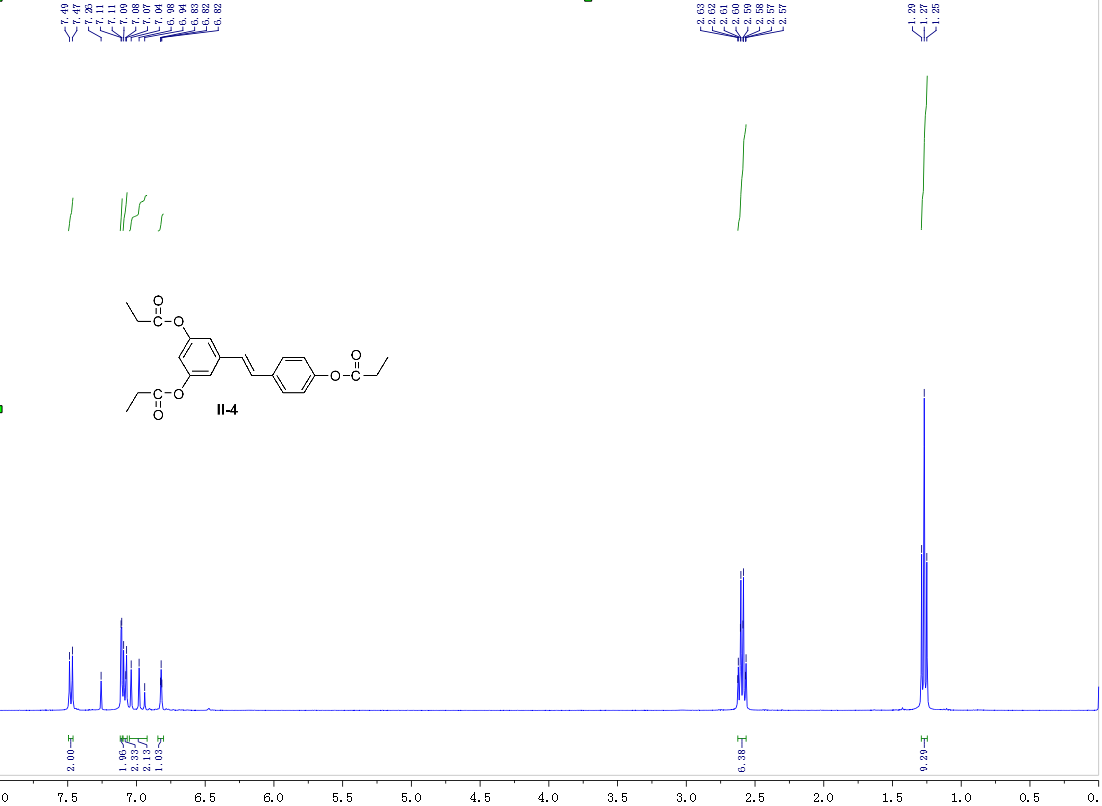


**Fig. 31** ^1^H NMR spectrum of **II-4**


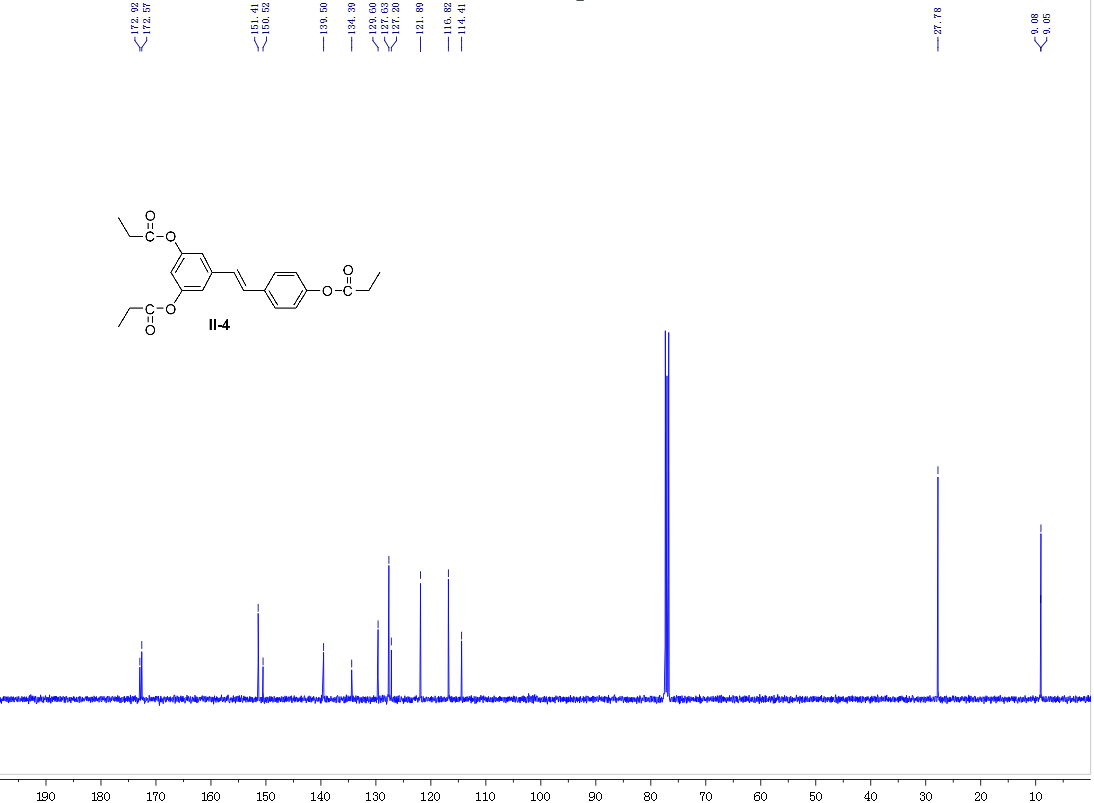


**Fig. 32** ^13^C NMR spectrum of **II-4**


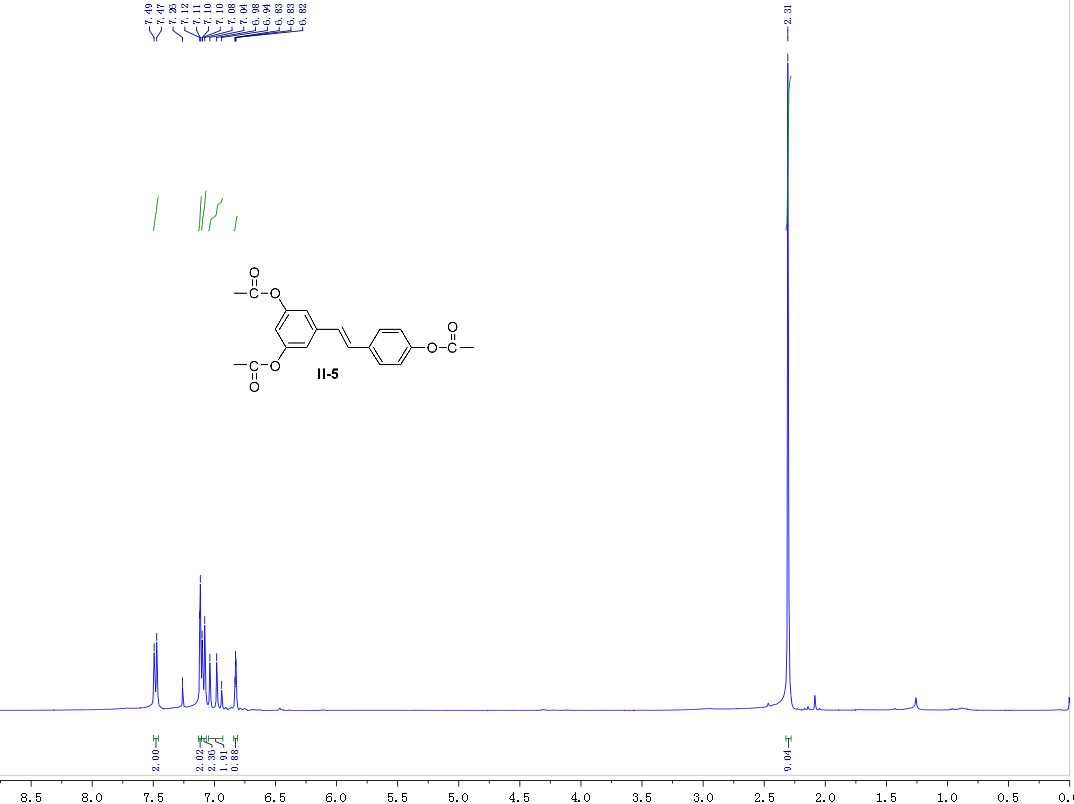


**Fig. 33** ^1^H NMR spectrum of **II-5**


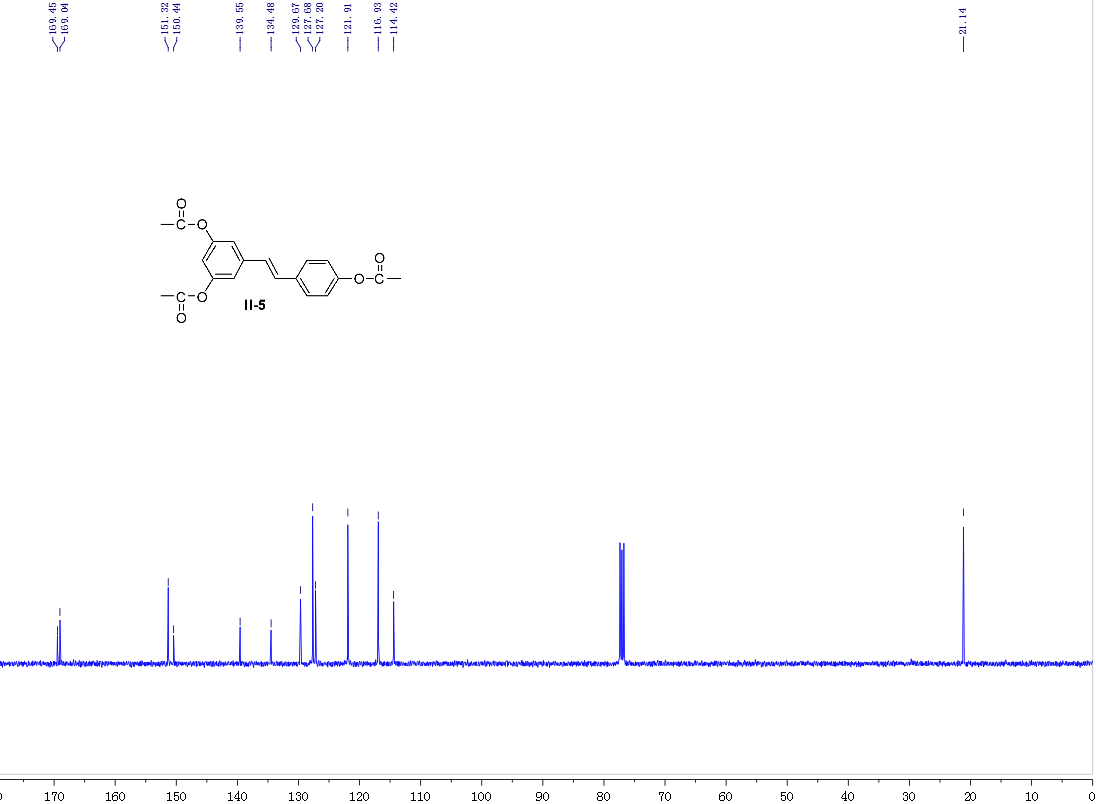


**Fig. 34** ^13^C NMR spectrum of **II-5**


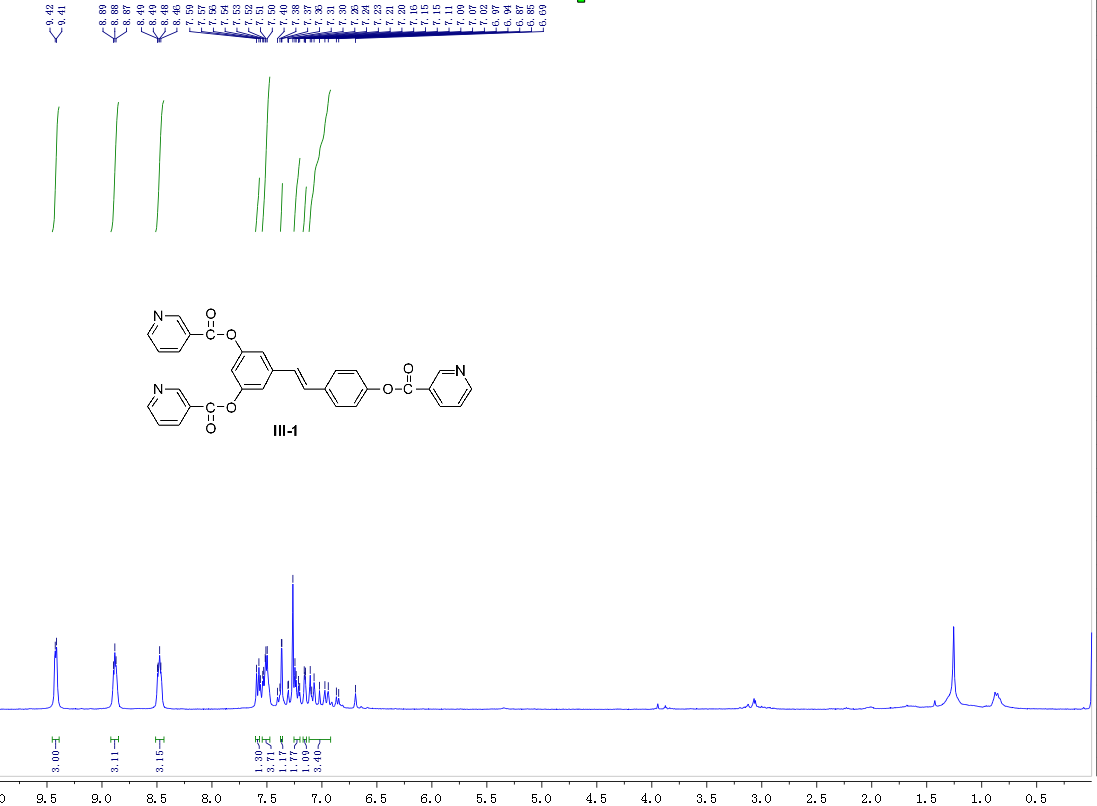


**Fig. 35** ^1^H NMR spectrum of **III-1**


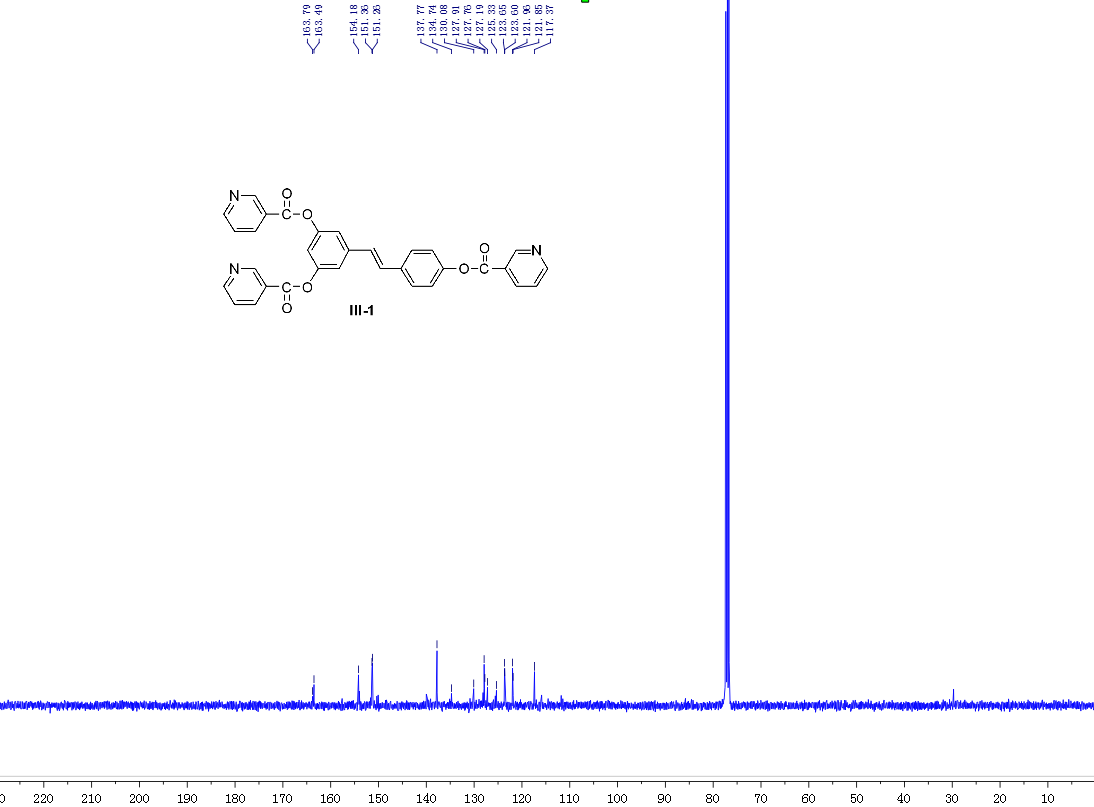


**Fig. 36** ^13^C NMR spectrum of **III-1**


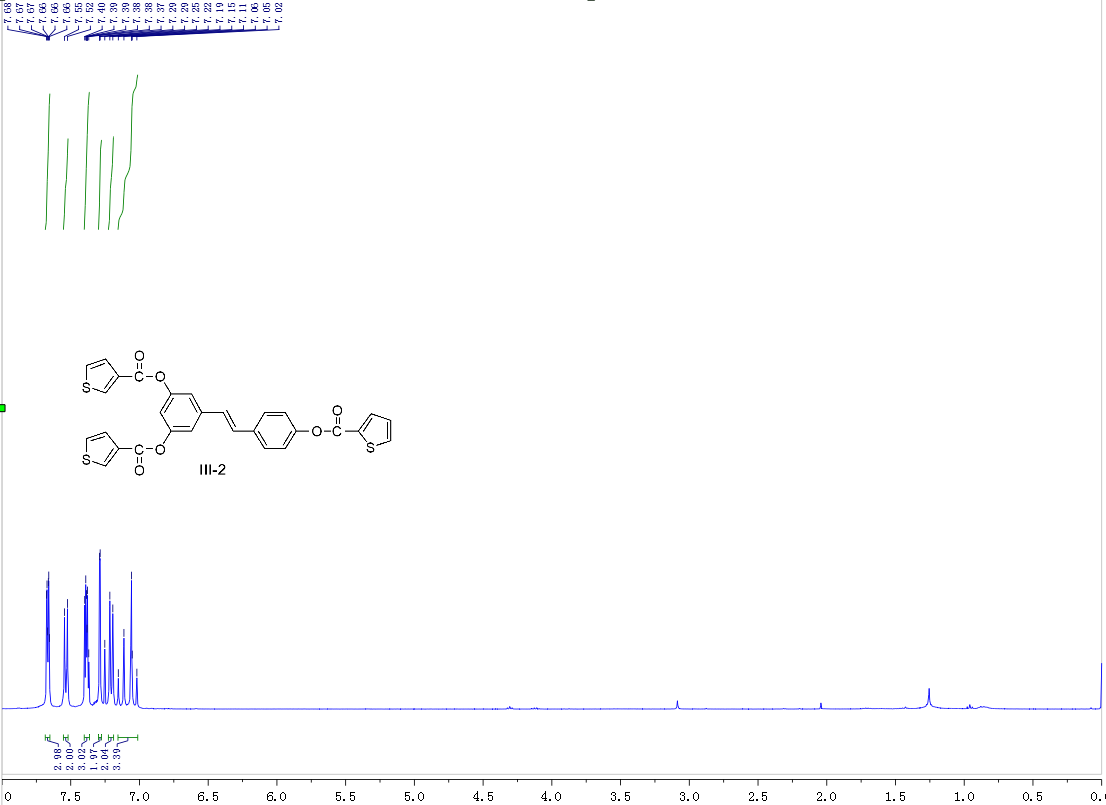


**Fig. 37** ^1^H NMR spectrum of **III-2**


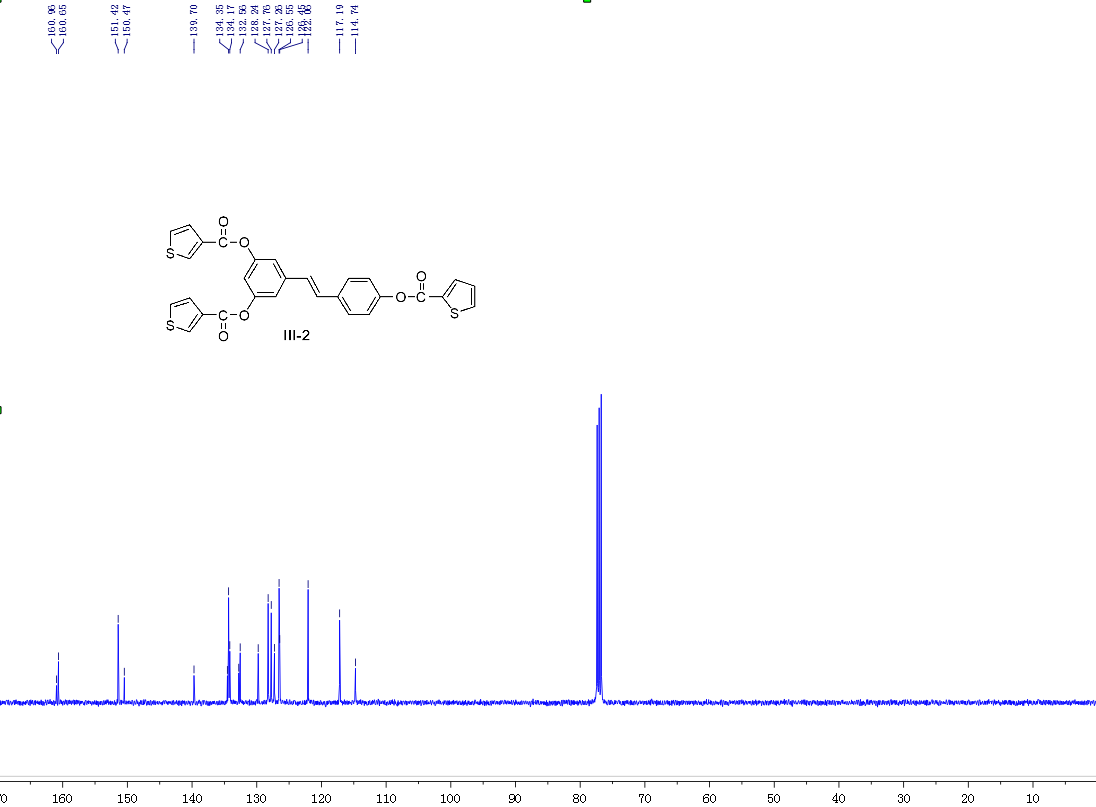


**Fig. 38** ^13^C NMR spectrum of **III-2**


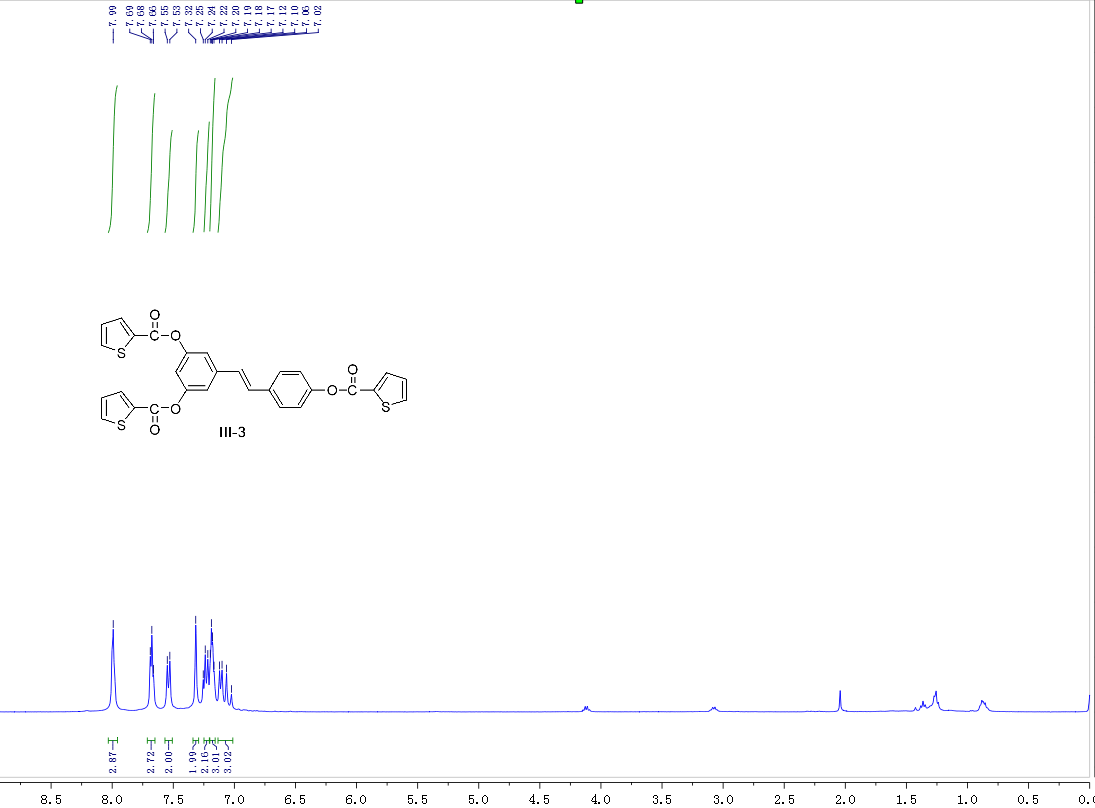


**Fig. 39** ^1^H NMR spectrum of **III-3**


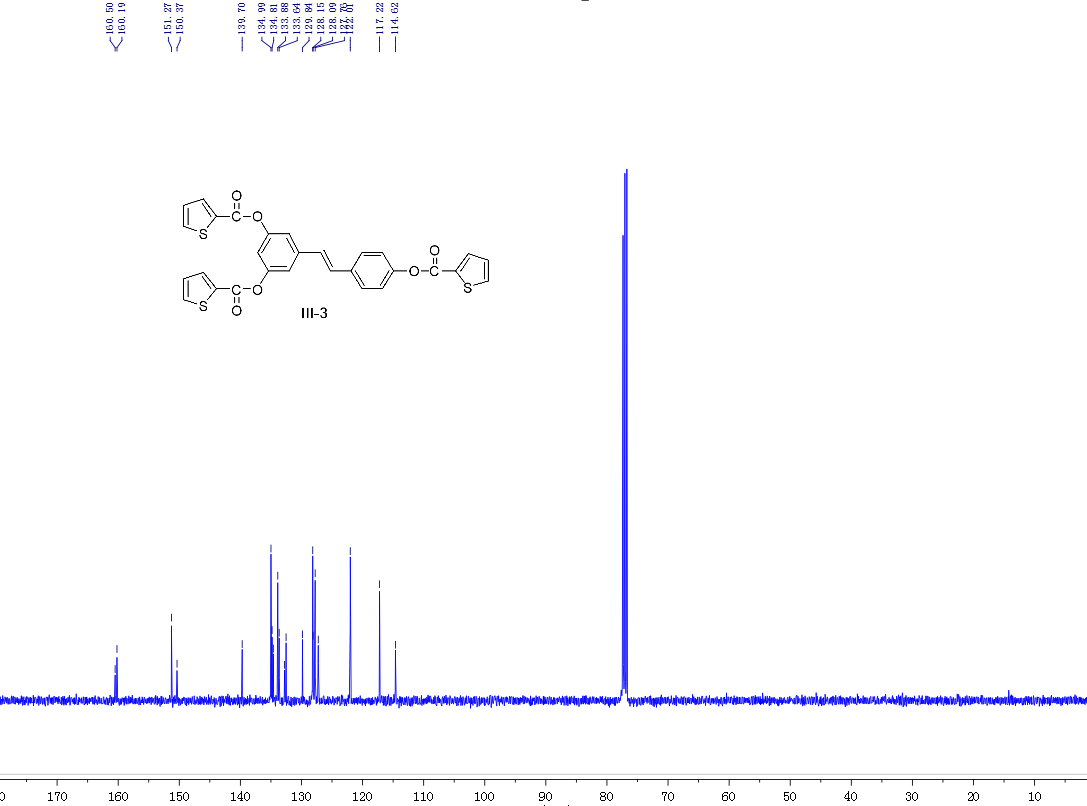


**Fig. 40** ^13^C NMR spectrum of **III-3**


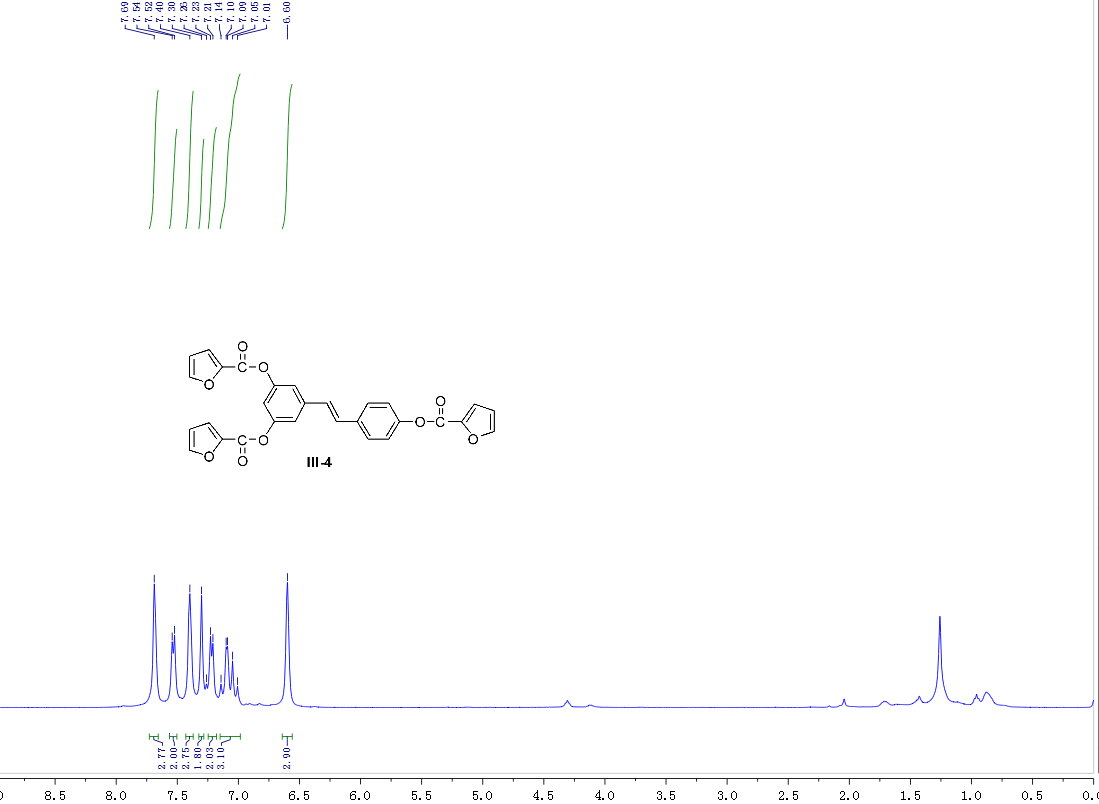


**Fig. 41** ^1^H NMR spectrum of **III-4**


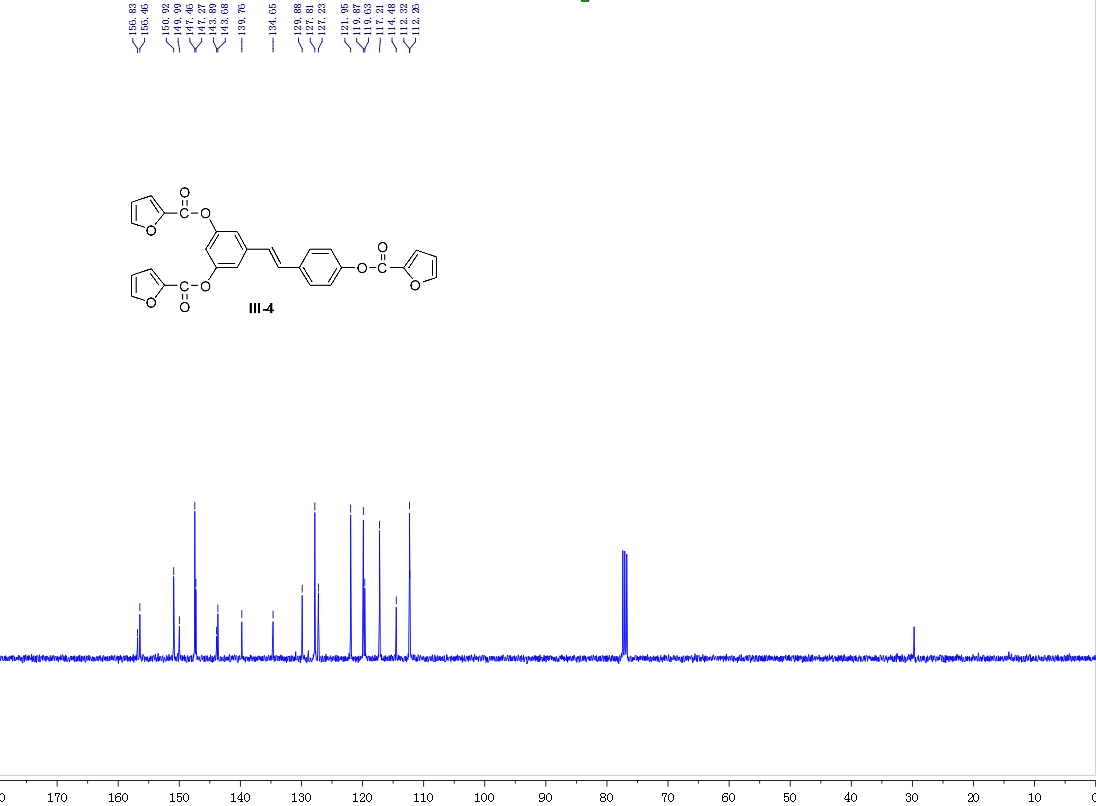


**Fig. 42** ^13^C NMR spectrum of **III-4**

**Biological assay**

***Detailed bioassay procedures for the anti-TMV activities.*** Compound solution preparation: Test compound was dissolved within a suitable amount of N,N-dimethyl formate and diluted with water containing 0.1% TW-80 to make a concentration of 500 μg/mL, and the aqueous solution was diluted to 100 μg/mL.

***Phytotoxic Activity.*** The phytotoxic activity test was carried out in a climate chamber at a temperature of 25 °C. The growing 3–5 leaf stage tobaccos (*Nicotiana tabacum var Xanthi nc*) were selected. The compound solution (100 μg/mL or 500 μg/mL) was sprayed on the leaves and then tested the plant height and weight changes after 0, 3, 7 and 10 days respectively. There are three replicates for each compound.

***Protective Effect of Compounds against TMV in Vivo.*** The compound solution was smeared on the growing *N. tabacum L.* leaves of the same ages. Another pot was smeared with solvent for control. After 12 h, the leaves were inoculated by the juice-leaf rubbing method and then washed with water. The local lesion numbers appearing 3−4 days after inoculation were counted. There were three replicates for each compound.

The juice-leaf rubbing method: Sprinkle emery (500 mesh) on the leaf surface, dip in the virus liquid with a brush, and rub the whole leaf surface along the branch vein twice. Support the underside of the leaf with the palm of the hand. The virus concentration is 10 µg/mL. After inoculation, rinse with running water.

***Inactivation Effect of Compounds against TMV in Vivo.*** The virus was inhibited by mixing with the compound solution at the same volume for 30 min. The mixture was then inoculated on the growing leaves of the same ages, whereas another pot was inoculated with the mixture of solvent and the virus for control. The local lesion numbers were recorded 3−4 days after inoculation. There were three replicates for each compound.

***Curative Effect of Compounds against TMV in Vivo****.* TMV (concentration of 6.0 × 10^−3^ mg/mL) was inoculated on the growing leaves of *N. tabacum L.* of the same ages. Then, the leaves were washed with water and dried. The compound solution was smeared on the leaves, whereas another pot was smeared with solvent for control. The local lesion numbers were then counted and recorded 3−4 days after inoculation. There were three replicates for each compound.

The in vitro and in vivo inhibition rates of the compound were then calculated according to the following formula (“av” means average, and controls were not treated with compound):

*inhibition rate (%) = [(av local lesion number of control − av local lesion number of drugtreated)/av local lesion number of control] × 100%.*

***Detailed bioassay procedures for the fungicidal activities.*** The compounds were evaluated in mycelial growth tests in artificial media against 14 plant pathogens at rate of 50 mg L^−1^. Test compound was dissolved within a suitable amount of acetone and diluted with water containing 0.1% TW-80 to the concentration of 500 mg L^-1^. To each petri dish was added 1 mL such solution and 9 mL culture medium to make a 50 mg L^-1^ of medicated tablet, whereas to another petri dish was added 1 mL sterilized water and 9 mL culture medium as blank control. A diameter of 4 mm of hyphae was cut by a hole puncher along the hyphae for bacteria to the outer plate and moved to the medicated tablet. Each treatment was performed three times. The dishes were stored in controlled environment cabinets (24±1°C) for 48 h, after which the diameter of mycelia growth was investigated and percentage inhibition was calculated.

*Percentage inhibition (%) = (averaged diameter of mycelia in blank controls – averaged diameter of mycelia in medicated tablets) / averaged diameter of mycelia in blank controls*

***Detailed bioassay procedures for the insecticidal activities.***

Larvicidal Activities against oriental armyworm (*Mythimna separata*), cotton bollworm (*Helicoverpa armigera*), corn borer (*Ostrinia nubilalis*):

Stock solutions of each test compound was prepared in dimethylformamide at a concentration of 600 mg L^-1^ and then diluted to the required concentration (200, 100, 50, 25, 10, 5, 2, and 1 mg L^-1^) with water containing TW-20.

Leaf-dip method was used. Leaf discs (5 cm × 3 cm) were cut from fresh cabbage leaves (or other leaves) and then dipped into the test solution for 3 s. After air-drying, the treated leaf discs were placed individually into vertical tube (or Petri dishes) and the discs were infested with 10 larvae (for example: 10 second-instar diamondback moth larvae, 10 fourth-instar oriental armyworm larvae). Percentage mortalities were evaluated 3 days after treatment.

Evaluations were based on a percentage scale of 0–100, where 0 equals no activity and 100 equals total kill. Each treatment was performed three times. Error of the experiments was about 5%.

***Larvicidal Activities against Mosquito (Culex pipiens pallens).*** 20 fourth-instar mosquito larvae were put into the 10 mL of the test solution. Percentage mortalities were evaluated 8 days after treatment.

Evaluations were based on a percentage scale of 0–100, where 0 equals no activity and 100 equals total kill. Each treatment was performed three times. Error of the experiments was about 5%.
